# Cathepsin B Regulates Ovarian Reserve Quality and Quantity via Mitophagy by Modulating IGF1R Turnover

**DOI:** 10.1111/acel.70066

**Published:** 2025-04-28

**Authors:** Aradhana Mohanty, Anjali Kumari, Lava Kumar. S, Ajith Kumar, Pravin Birajdar, Rohit Beniwal, Mohd Athar, Kiran Kumar. P, H.B.D.Prasada Rao

**Affiliations:** ^1^ BRIC‐National Institute of Animal Biotechnology Hyderabad Telangana India; ^2^ Graduate Studies Regional Center for Biotechnology Faridabad India

**Keywords:** autophagy, cathepsin B, IGF1R, mitophagy, ovarian reserve

## Abstract

The quality and quantity of the ovarian reserve are meticulously regulated through various cell death pathways to guarantee the availability of high‐quality oocytes for fertilization. While apoptosis is recognized for contributing to maintaining ovarian reserve, the involvement of other cell death pathways remains unclear. Employing chemical genetics and proteomics, this study reveals the crucial involvement of Cathepsin B in maintaining the ovarian reserve. Results indicate that apoptosis and autophagy play pivotal roles, and inhibiting these pathways significantly increases follicle numbers. Proteomics reveals a dynamic shift from apoptosis to autophagy during follicular development, with Cathepsin B emerging as a key player in this transition. Inhibiting Cathepsin B not only mimics the augmented oocyte reserve observed with autophagy inhibition but also upregulated IGF1R and AKT–mTOR pathways without compromising fertility in pre‐ and postpubertal mice. Further, IGF1R inhibition partially compromised the protective effects of Cathepsin B inhibition on oocyte reserves, suggesting their interdependence. This association is further supported by the finding that Cathepsin B can degrade IGF1R in vitro. Moreover, the increased IGF1R levels enhance the oocyte mitochondrial membrane potential via transcriptional regulation of mitochondrial biogenesis and mitophagy genes. Remarkably, this Cathepsin B‐dependent ovarian reserve maintenance mechanism is conserved in higher‐order vertebrates. Cumulatively, our study sheds valuable light on the intricate interplay of autophagy, Cathepsin B, and growth factors in ovarian reserve maintenance, offering potential therapeutic strategies to delay ovarian aging and preserve fertility.

## Graphical abstract

Autophagy regulates ovarian reserve, with Cathepsin B playing a critical role in aging. Inhibition of Cathepsin B increases follicle count, enhances IGF1R–AKT–mTOR signaling and improves mitochondrial function without compromising fertility. This conserved mechanism suggests potential therapeutic strategies to delay ovarian aging and sustain fertility, offering new insights into reproductive longevity.

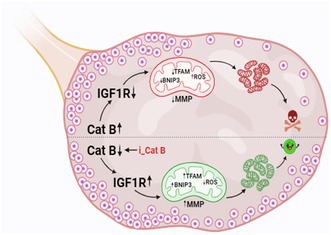

## 1 Introduction

Throughout a female's reproductive lifespan, regulated cell death mechanisms play fundamental roles at various stages of ovarian development, like follicle formation, development, ovulation, and eliminating damaged oocytes (Yadav et al. 2018; Grive 2020; Stringer et al. 2023; Vaskivuo and Tapanainen 2003; Hussein 2005; Regan et al. 2018; Krysko et al. 2008; Singh et al. 2023). For example, in humans, the number of oocytes follows a continuous decreasing trend from a peak of 6–7 million oocytes at 20 weeks of gestation to less than 1 million at birth. As a girl reaches menarche, the number further decreases to approximately 300,000–400,000 oocytes (Krysko et al. 2008; Wilkosz et al. 2014; Findlay et al. 2015; Hansen et al. 2008; Faddy 2000; Perez et al. 2000). During the reproductive years, the rate of decline remains relatively steady at about 1000 follicles per month, but this decline accelerates significantly after the age of approximately 37 years. Ultimately, at menopause, the number of remaining follicles drops below 1000, indicating that ovarian reserve dictates the reproductive lifespan in females (Wilkosz et al. 2014; Findlay et al. 2015; Faddy 2000). This pattern of oocyte attrition is not exclusive to humans and is conserved across different species (Singh et al. 2023). While the loss of germ cells is ongoing throughout a female's life, significant loss occurs with early oocytes called primordial and primary follicles (Vaskivuo and Tapanainen 2003; Findlay et al. 2015). It is widely acknowledged that oocyte apoptotic cell death processes cause atresia in primordial and primary follicles via the DNA damage response pathway (DDR) (Singh et al. 2023, 2021). DDR in early oocytes before birth resembles that observed in somatic cells. It involves the initiation of checkpoint signaling through activating ATM/ATR kinases, which in turn phosphorylate downstream targets to enhance DDR signaling, support damage repair, or trigger programmed cell death. Following birth, primordial follicles constitutively express an alternative member of the p53 family, TAp63, making them highly susceptible to damage‐induced apoptosis (Qiao et al. 2018; Luan et al. 2022; Bolcun‐Filas et al. 2014; Livera et al. 2008; Tuppi et al. 2018).

On the other hand, active autophagy was observed in perinatal ovaries consisting of very early oocytes (Zhihan et al. 2019). Inhibition of autophagy increased the number of cyst oocytes and delayed folliculogenesis in organ cultures (Zhihan et al. 2019). In addition, depletion of Becn1 or Atg7 resulted in elevated germ cell loss and subfertility, indicating the roles of other cell death pathways in oocyte reserve maintenance (Gawriluk et al. 2011). Similarly, microRNA let‐7 g controls autophagy in mouse granulosa cells by targeting the insulin‐like growth factor 1 receptor (IGF1R) and inhibiting the mammalian target of the rapamycin signaling pathway (Zhou et al. 2016). Specifically, the quality of oocytes is preserved with age in *C. elegans* through the mutation of the *daf‐2* insulin/insulin‐like growth factor signaling (IIS) receptor, a process influenced by Cathepsin B activity (Templeman et al. 2018). Cathepsins are a group of protease enzymes involved in the final stages of autophagy (Xie et al. 2023). Among them, Cathepsin B is a cysteine protease primarily responsible for protein degradation (Xie et al. 2023). Previous studies have shown that Cathepsins B, C, and S are upregulated in cancerous chicken ovaries, suggesting their potential roles in ovarian cancer development (Ahn et al. 2010). Additionally, inhibition of Cathepsin B in vitro cultures has been shown to improve oocyte and blastocyst quality (Pezhman et al. 2017; Li et al. 2020). However, the significance of alternative death pathways and their mechanisms in early follicular atresia is unknown. This study shows that inhibition of lysosomal cysteine protease Cathepsin B enhances the oocyte reserve via regulating IGF1R turnover.

## 2 Results

### 2.1 Postnatally, Both Apoptosis and Autophagy Play Integral Roles in Preserving Ovarian Reserve

**FIGURE 1:**
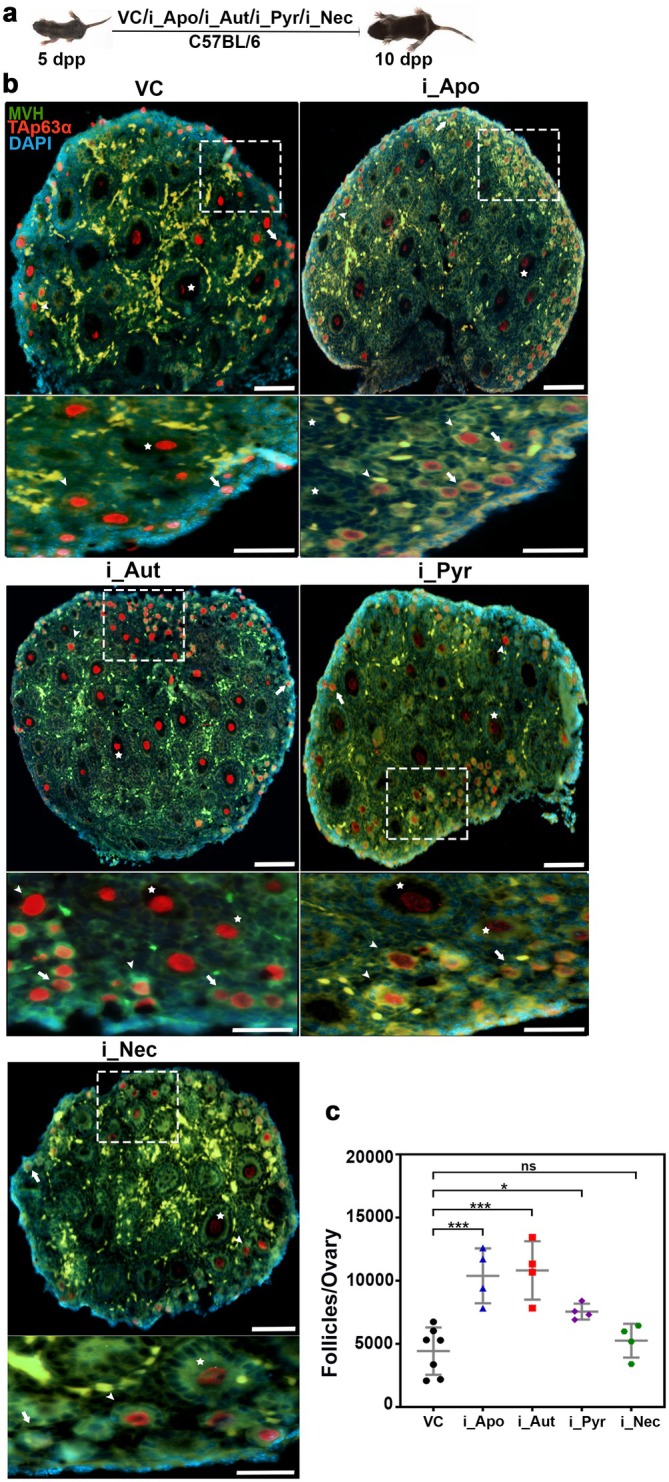
Role of death pathways in ovarian reserve maintenance. (a) Intraperitoneal injection of death pathway inhibitors: An experimental regimen. (b) Ten days postpartum (dpp) ovary sections from VC‐, i_Apo‐, i_Aut‐, i_Pyr‐, and i_Nec‐treated females immunostained for TAp63α (red), MVH (green), and DNA (blue). (c) Follicle counts at 10 dpp from VC‐, i_Apo‐, i_Aut‐, i_Pyr‐, and i_Nec‐treated females. VC, vehicle control; i_Apo, an inhibitor of apoptosis; i_Aut, an inhibitor of autophagy; i_Pyr, an inhibitor of pyroptosis; i_Nec, an inhibitor of necrosis. Arrows, arrowheads, and stars highlight the primordial, primary, and secondary follicles, respectively. The zoomed images adjacent to the ovaries are represented with white squares. ***p ≤ 0.001, *p ≤ 0.01, ns ≥ 0.1 unpaired *t*‐test. Error bars show mean ± SD. Scale bars for ovary sections are 100 μm. The animals used *n* = 3–4.

In exploring the fundamental pathways contributing to early ovarian follicle depletion, we thoroughly investigated specific inhibitors administered to 5‐day‐old female mice. This particular stage was chosen due to the abundance of early follicles, allowing for the administration of inhibitors without causing harm to the mice. The experimental cohorts were subjected to a dosage of 5 mg/kg body weight for an apoptosis inhibitor (Z‐VAD‐FMK) 50 mg/kg for an autophagy inhibitor (3‐MA), 10 mg/kg for a pyroptosis inhibitor (Disulfiram), and 5 mg/kg for a necrosis inhibitor (GSK^’^ 872), with a single dose of alternate day administration (Figure 1a) (Equils et al. 2009; Li et al. 2018; Bernier et al. 2020; Zhou et al. 2019). Ovaries were harvested on the 10th day, subsequently fixed, sectioned, and subjected to immunostaining for the germ cell‐specific marker, mouse vasa homolog (MVH), and the guardian of germ cells, TAp63α (Figure 1b). Analysis of follicle counts unveiled that mice treated with apoptosis, autophagy, and pyroptosis inhibitors exhibited 2.4‐, 2.4‐, and 1.7‐fold increases in ovarian follicle numbers, respectively, compared to the vehicle control group (Figure 1c and Figure S1a–c). In contrast, mice treated with the necrosis inhibitor displayed no significant variations, suggesting that apoptosis and autophagy hold equal importance in the early loss of ovarian follicles, whereas pyroptosis may have a secondary impact (Figure 1c and Figure S1a–c). Notably, the coadministration of inhibitors targeting apoptosis and autophagy from 5 to 10 days postpartum (dpp), or exclusively using inhibitors for apoptosis, autophagy, pyroptosis, and necrosis within a 5–21‐day timeframe, resulted in lethality in mice, underscoring the intricate balance these pathways uphold for the overall health of the animals.

### 2.2 Autophagy and Lysosomal Cysteine Proteases Are Upregulated During Folliculogenesis

**FIGURE 2:**
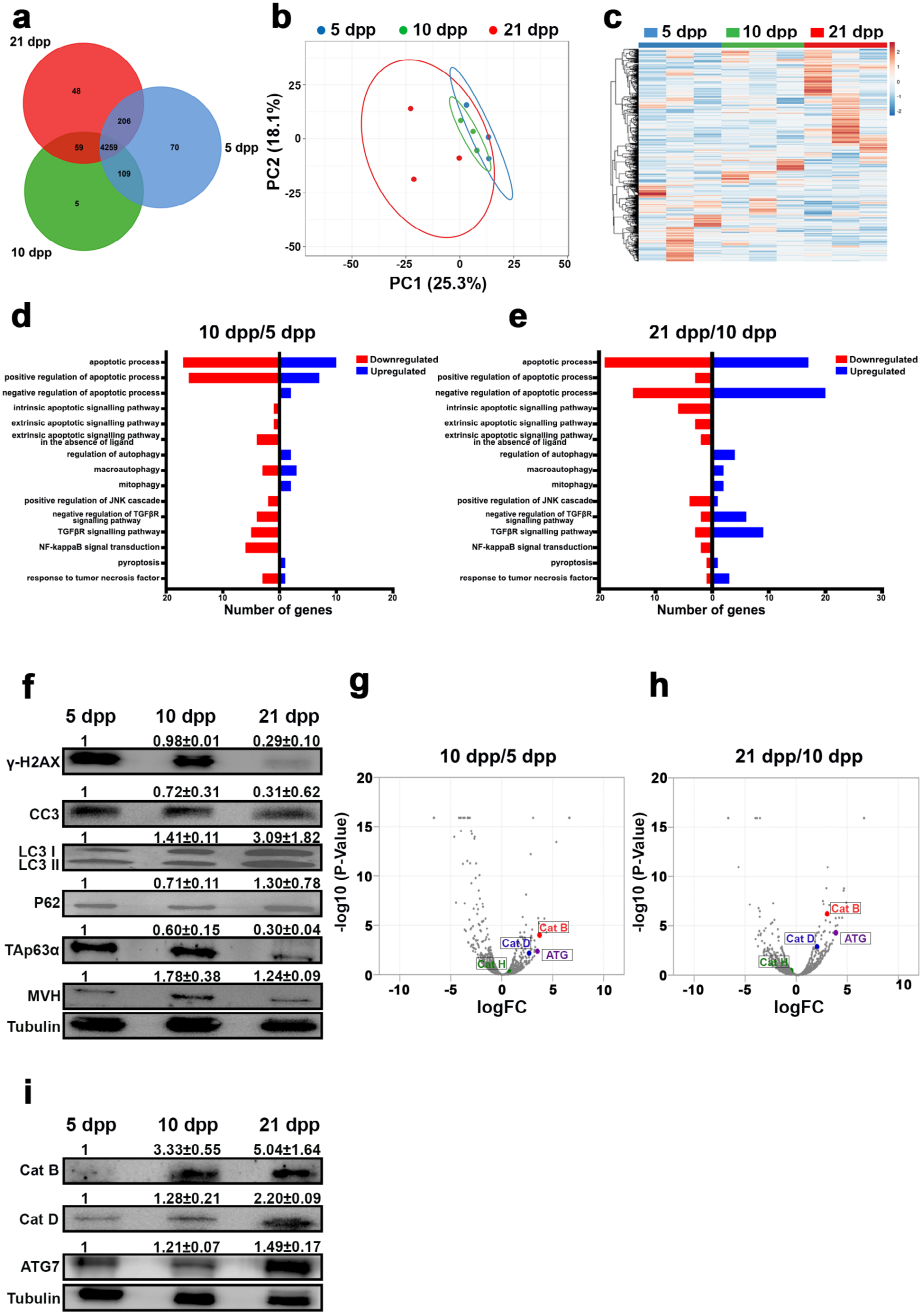
Proteomic profiling of mice ovary in different age groups. (a) Venn diagram of 5, 10, and 21 dpp mice ovaries proteome (*n* = 3). (b) Principal component analysis (PCA) shows the difference between the 5, 10, and 21 dpp proteome profiles. (c) The relative abundance of differential proteins in 5, 10, and 21 dpp is presented as a heat map. (d) and (e) Distribution of death pathways related proteins identified in 5 and 10 dpp ovaries, 10 and 21 dpp ovaries, respectively. (f) Western blot analysis of γH2AX, cc3, LC3, P62, TAp63α, MVH, and Tubulin from 5, 10, and 21 dpp ovaries. (g) and (h) A volcano plot showing *p* values versus fold changes of all proteins in 5 and 10 dpp ovaries, 10 and 21 dpp ovaries, respectively. (i) Western blot analysis of Cat B, Cat D, ATG7, and Tubulin from 5, 10, and 21 dpp ovaries.

To comprehend the intricacies of death pathways in folliculogenesis, we conducted untargeted proteomics on ovaries from mice at different developmental stages (5, 10, and 21 days postpartum). The results revealed the identification of 4644, 4432, and 4572 proteins in 5‐, 10‐, and 21‐day‐old ovaries (*n* = 3). Intriguingly, 70, 5, and 48 proteins were uniquely identified in 5‐, 10‐, and 21‐day‐old ovaries, respectively, while 4259 proteins were common across all samples (Figure 2a). Multivariate discriminative analysis unveiled a significant separation between the three developmental stages with 25.3% PC1 and 18.1% PC2 contributing to the distinction (Figure 2b). The generated heat map and hierarchical clustering analysis demonstrated distinct clusters corresponding to 5‐, 10‐, and 21‐day‐old ovaries (Figure 2c). Delving into the biological functions of the identified proteins, we observed a downregulation of apoptosis‐related genes in 10‐day‐old ovaries compared to 5‐day‐old ovaries. Concurrently, upregulation of autophagy‐related genes in 10‐day‐old ovaries suggested the activation of autophagy during this developmental phase, coinciding with decreased apoptosis activity (Figure 2d). A similar comparison between 21‐ and 10‐day‐old ovaries indicated an upregulation of autophagy‐related genes, signifying heightened autophagic activity in 21 dpp (Figure 2e). The balanced regulation of apoptosis genes in 21‐day‐old ovaries compared to 10‐day‐old ovaries suggested intricate dynamics, possibly influenced by other somatic cells like cumulus cells (Figure 2d,e). Corroborating the proteomic findings, Western blot analyses validated reduced DNA damage levels (γH2AX), diminished apoptosis levels (cc3), decreased oocyte reserve (TAp63α), and increased autophagy levels (LC3 and P62) in ovaries at 5, 10, and 21 days postpartum (Figure 2f). Notably, the elevated autophagy levels during folliculogenesis prompted an in‐depth exploration of the abundance of proteins at 5 dpp compared to 10 dpp and 10 dpp compared to 21 dpp. Among the top 50 most abundant proteins, significant findings included the presence of cysteine proteases Cathepsins B and D and the autophagy protein ATG7 in ovaries at both 10 and 21 days of age (Figure 2g,h). Subsequent validation through Western blots conclusively confirmed a discernible upregulation of lysosomal cysteine proteases and autophagy during folliculogenesis (Figure 2i).

### 2.3 Cathepsin B Inhibition Protects the Ovarian Reserve

**FIGURE 3:**
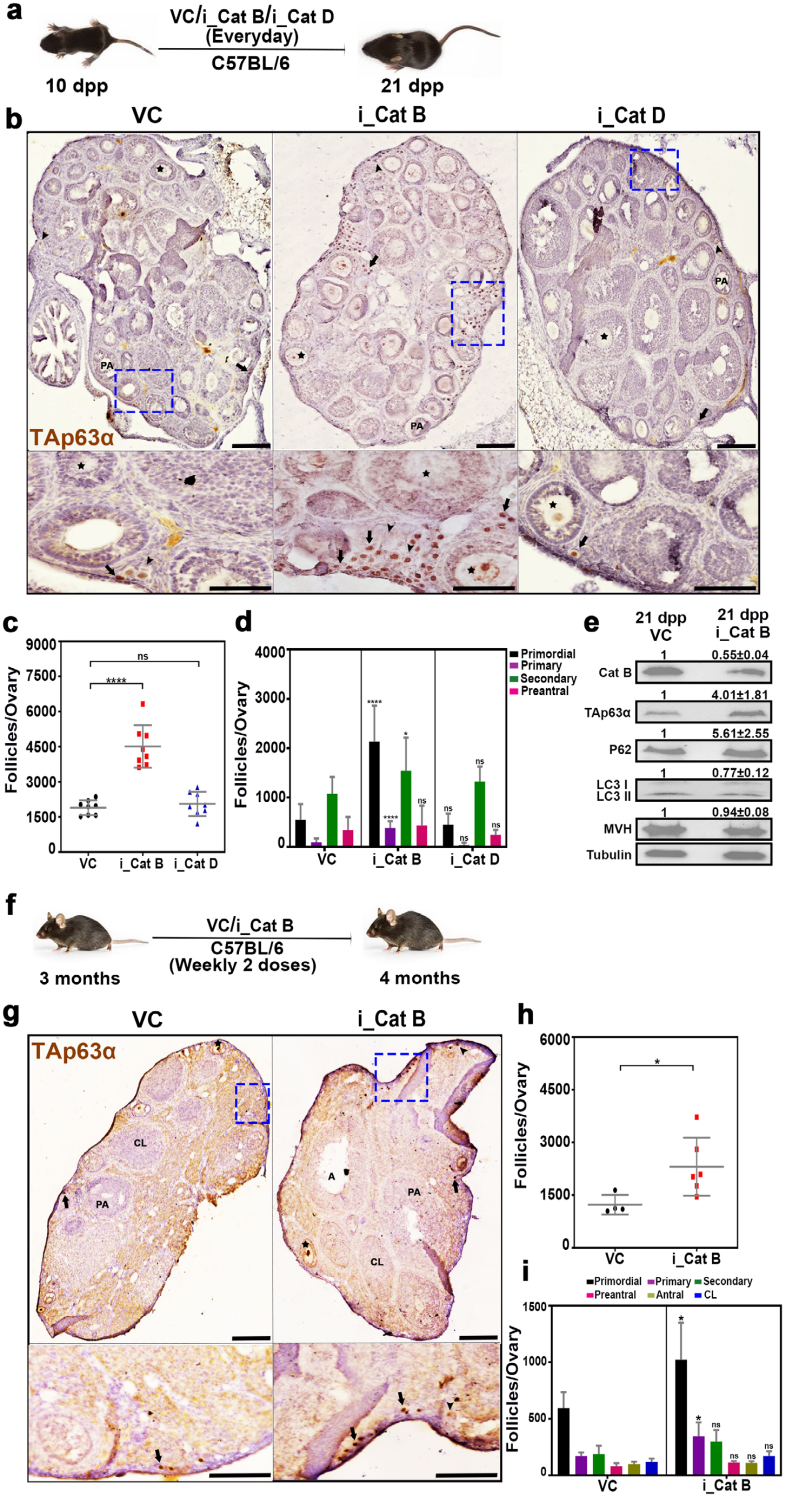
Inhibition of Cathepsin B protects the ovarian reserve. (a) Experimental regime for intraperitoneal injection of Cathepsin B and D inhibitors in prepubertal mice (*n* = 4). (b) Twenty‐one days postpartum ovary sections from VC‐, i_Cat B‐, and i_Cat D‐treated females immunostained for TAp63α with hematoxylin counterstaining. (c) Follicle counts at 21 dpp from VC‐, i_Cat B‐, and i_Cat D‐treated females. (d) Types of follicles from 21 dpp from VC‐, i_Cat B‐, and i_Cat D‐treated females. (e) Western blot analysis of Cat B, TAp63α, P62, LC3, MVH, and Tubulin from VC‐ and i_Cat B‐treated ovaries. (f) Experimental regime for intraperitoneal injection of Cathepsin B inhibitor in postpubertal mice (*n* = 3). (g) Four months ovary sections from VC‐ and i_Cat B‐treated females immunostained for TAp63α with hematoxylin counterstaining. (h) Follicle counts at 4 months from VC‐, i_Cat B‐, and i_Cat D‐treated females. (i) Types of follicles from 4 months from VC‐ and i_Cat B‐treated females. VC, vehicle control; i_Cat B, an inhibitor of Cathepsin B; i_Cat D, an inhibitor of Cathepsin D; PA, preantral follicle; A, antral follicle; CL, corpus luteum. Arrows, arrowheads, and stars highlight the primordial, primary, and secondary follicles, respectively. The zoomed images adjacent to the ovaries are represented with blue squares. ****p ≤ 0.0001, *p ≤ 0.05, ns ≥ 0.1 unpaired *t*‐test. Error bars show mean ± SD. The significance *p* values shown in (d) and (i) represent comparisons between the control group (VC) and the treatment groups (i_Cat B or i_Cat D). Scale bars for ovary sections are 100 μm.

Given the abundance of Cathepsins B and D in mouse ovaries at 10 and 21 days postpartum, and the findings from *C. elegans* research indicating that the insulin‐signaling pathway regulates oocyte quality through Cathepsin B, an enzyme critical for granulosa cell proliferation, we investigated the effects of inhibiting Cathepsins B and D on ovarian reserves (Templeman et al. 2018; Chen et al. 2021). To this end, we administered Myricetin, a Cathepsin B inhibitor, at a dose of 37.5 mg/kg body weight, and Pepstatin A, a Cathepsin D inhibitor, at 20 mg/kg body weight, daily to 10‐day‐old female mice. Ovaries were then collected on day 21 (Figure 3a) (Fox et al. 2016; Ramalho et al. 2015). The ovaries were then fixed, sectioned, and immunostained for TAp63α (Figure 3b). Follicle counts revealed that Cathepsin B‐inhibited ovaries had 2.4 times more follicles compared to control ovaries, whereas Cathepsin D inhibition did not result in a significant increase in follicle numbers (Figure 3c). Further analysis showed an increase in early‐stage follicles, including primordial, primary, and secondary follicles, in the Cathepsin B‐inhibited ovaries (Figure 3d). Additionally, the protective effect of Cathepsin B inhibition on follicle count was found to be dose‐dependent (Figure S2b). Western blot analysis confirmed a 45% and 33% reduction in the protein levels of Cathepsin B and LC3, respectively, following Cathepsin B inhibition. In contrast, TAp63α levels increased fourfold, suggesting that Cathepsin B inhibition is protective in maintaining the ovarian reserve (Figure 3e).

To evaluate the effect of Cathepsin B inhibition on the follicular reserve in postpubertal mice, we administered Myricetin at 37.5 mg/kg body weight to 3‐month‐old female mice weekly with a double dose, collecting ovaries at 4 months (Figure 3f). Similar to the prepubertal treatment, Cathepsin B inhibition in postpubertal mice resulted in a 1.9‐fold increase in follicle numbers compared to controls, with an increase in primordial and primary follicles observed (Figure 3g–i). To determine if the protected oocytes could undergo successful maturation and embryogenesis, mice treated with the Cathepsin B inhibitor from 10 to 21 dpp were sacrificed on the 60th day (Figure S2d). Serum and oocytes were collected for endocrine profiling and oocyte quality assessment (Figure S2d). Results showed no significant differences in oocyte maturation and embryogenesis, indicating that the protected oocytes were of high quality (Figure S2f–i). Endocrine profiling revealed no significant effects from Cathepsin B inhibition (Figure S2e). Furthermore, a cohort of treated animals was monitored for up to 8 litters, and no differences in litter size or fertility were observed compared to controls, indicating that Cathepsin B inhibitor treatment does not affect fertility (Figure S2j). To evaluate the sustained protective effects of Cathepsin B inhibition on ovarian reserve, we employed a daily administration of the Cathepsin B inhibitor from postnatal days 10–21, followed by a 6‐month observation period (Figure S2a). Subsequently, ovaries were collected, fixed, sectioned, and subjected to immunostaining for TAp63α. Examination of follicular quantification revealed a 1.9‐fold increase in the ovaries treated with the Cathepsin B inhibitor compared to the control group (Figure S2c). This increase was predominantly observed in enhanced primordial and primary follicles, suggesting that early Cathepsin B inhibition may contribute to the long‐term protection of the follicular reserve.

Furthermore, in assessing the genetic prerequisites for early follicle protection through Cathepsin B inhibition, we conducted coadministration experiments with inhibitors targeting apoptosis, autophagy, pyroptosis, or necrosis from 5 to 10 days dpp (Figure S3a). Ovaries were collected on the 10th day, fixed, sectioned, and subjected to immunostaining for MVH and TAp63α. Our analysis revealed a reduction in follicle numbers when the Cathepsin B inhibitor was coadministered with the apoptosis inhibitor, in contrast to the use of the Cathepsin B or apoptosis inhibitor alone, suggesting an independent functioning of the two pathways and lethality to follicles when both death pathways are inhibited. Similarly, coadministration of the Cathepsin B inhibitor with the autophagy inhibitor replicated the effects of autophagy inhibitor treatment, indicating the involvement of Cathepsin B in regulating the ovarian reserve through the autophagy pathway. No significant difference in follicle numbers was observed when the Cathepsin B inhibitor was coadministered with the pyroptosis inhibitor, suggesting redundant roles in this context. Finally, coadministration of the Cathepsin B inhibitor with the necrosis inhibitor resulted in a decrease in follicle numbers compared to Cathepsin B alone, indicating a potential role of necrosis in Cathepsin B‐inhibited follicles (Figure S3b).

### 2.4 Cathepsin B Regulates IGF‐1R Turnover

**FIGURE 4:**
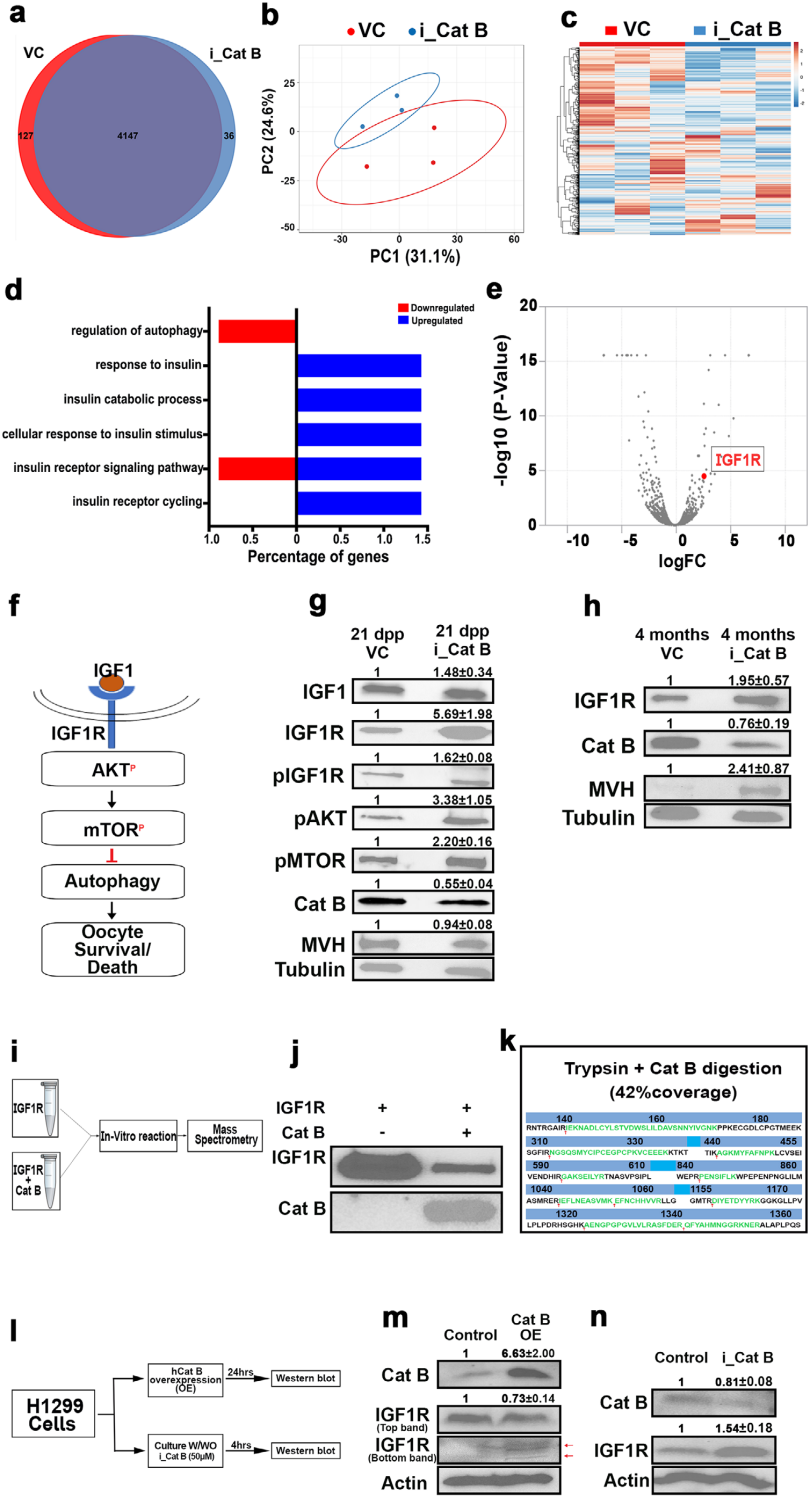
Proteomic profiling of Control and i_Cat B treated mice ovaries. (a) Venn diagram of VC‐ and i_Cat B‐treated ovaries proteome (*n* = 3). (b) Principal component analysis (PCA) shows the difference between the VC‐ and i_Cat B‐treated ovaries proteome profiles. (c) Relative abundance of differential proteins in VC‐ and i_Cat B‐treated ovaries presented as a heat map. (d) Distribution of all protein classes identified in VC‐ and i_Cat B‐treated ovaries according to biological process. (e) A volcano plot showing *p* values versus fold changes of all proteins in VC‐ and i_Cat B‐treated ovaries. (f) Schematic representation of insulin signaling pathway in oocytes. (g) Western blot analysis of IGF1, IGF1R, pIGF1R, pAKT, pMTOR, Cat B, MVH, and Tubulin from 21 dpp VC‐ and i_Cat B‐treated ovaries. (h) Western blot analysis of IGF1R, Cat B, MVH, and Tubulin from 4 months VC‐ and i_Cat B‐treated ovaries. (i) Experimental regimen for in vitro reaction followed by mass spectrometry. (j) Western blot analysis of in vitro digestion by Cat B. (k) Schematic representation of in vitro digested peptides of IGF1R identified in mass spectrometry. (l) Experimental regimen for H1299 cell culture experiments. (m) Western blot analysis of Cat B, IGF1R, and Actin from Control and Cat B‐overexpressed H1299 cells. (n) Western blot analysis of Cat B, IGF1R, and Actin from Control and i_Cat B‐treated H1299 cells. VC, vehicle control; i_Cat B, an inhibitor of Cathepsin B.

Besides, we conducted untargeted proteomics on ovaries from 21‐day‐old control and Cathepsin B inhibitor‐treated mice ovaries to elucidate the protective mechanism of Cathepsin B inhibition on the ovarian reserve. Our analysis revealed the identification of 4274 and 4183 proteins in control and Cathepsin B‐inhibited ovaries, respectively (*n* = 3). Notably, 127 and 36 proteins were found exclusively in control and Cathepsin B‐inhibited ovaries, whereas 4147 proteins were shared between both groups (Figure 4a). Multivariate discriminative analysis highlighted a significant separation between the two samples, with 31.1% PC1 and 24.6% PC2 contributing to this distinction (Figure 4b). The subsequent heat map and hierarchical clustering analysis confirmed distinct clusters corresponding to control and Cathepsin B‐inhibited ovaries (Figure 4c). Examining the biological functions of the identified proteins, we observed a downregulation of autophagy‐related genes and an upregulation of insulin‐regulatory genes in Cathepsin B‐inhibited ovaries compared to the control (Figure 4d). Elevated insulin regulatory pathways in ovaries with inhibited Cathepsin B prompted a thorough investigation into protein abundance. Among the top 50 most abundant proteins, IGF1R was notably present (Figure 4e). Western blot analyses confirmed increased levels of IGF1, IGF1R, and AKT pathway proteins, supporting the proteomic results and indicating that Cathepsin B influences insulin‐signaling pathways (Figure 4g). Furthermore, increased IGF1R levels were observed in postpubertal mice treated with a Cathepsin B inhibitor, underscoring Cathepsin B's significant role in regulating IGF1R in these mice (Figure 4h).

Specifically, we observed a 5.7‐fold increase in IGF1R levels in prepubertal ovaries and a twofold increase in postpubertal ovaries treated with Cathepsin B, which led us to hypothesize that IGF1R may be a substrate for the cysteine protease Cathepsin B (Figure 4g–h). Further, immunostaining of Cathepsin B and IGF1R across the two experimental groups revealed a decrease in Cathepsin B intensity and an increase in IGF1R intensity in the treated group (Figure S7g–m). To explore this hypothesis, we conducted an in vitro reaction involving human recombinant Cathepsin B incubated with recombinant IGF1R. Subsequently, we quenched the reaction and subjected it to western blot and mass spectrometry analysis (Figure 4i). The western blot results indicated a reduction in IGF1R levels in the presence of Cathepsin B (Figure 4j). Mass spectrometry analysis further revealed that IGF1R treated with Cathepsin B and trypsin displayed 10 specific digestion sites, in contrast to trypsin alone (Figure 4k). To validate whether IGF1R is indeed a substrate for Cathepsin B, we overexpressed human Cathepsin B in H1299 cells (Figure 4l). After 24 h, analysis of Cathepsin B and IGF1R levels through western blot suggested that the overexpression of Cathepsin B led to a decrease in IGF1R levels, accompanied by observation of specific degraded bands compared to the control (Figure 4m). Conversely, when a Cathepsin B inhibitor was added to H1299 cells and harvested after 4 h, western blot analysis showed increased IGF1R levels (Figure 4n). These in vitro and in vivo results suggest that inhibiting Cathepsin B leads to the accumulation of IGF1R, supporting the notion that IGF1R may act as a substrate for Cathepsin B.

### 2.5 The Inhibition of IGF1R Partially Mitigates the Effect of Cathepsin B Inhibition on the Ovarian Reserve

**FIGURE 5:**
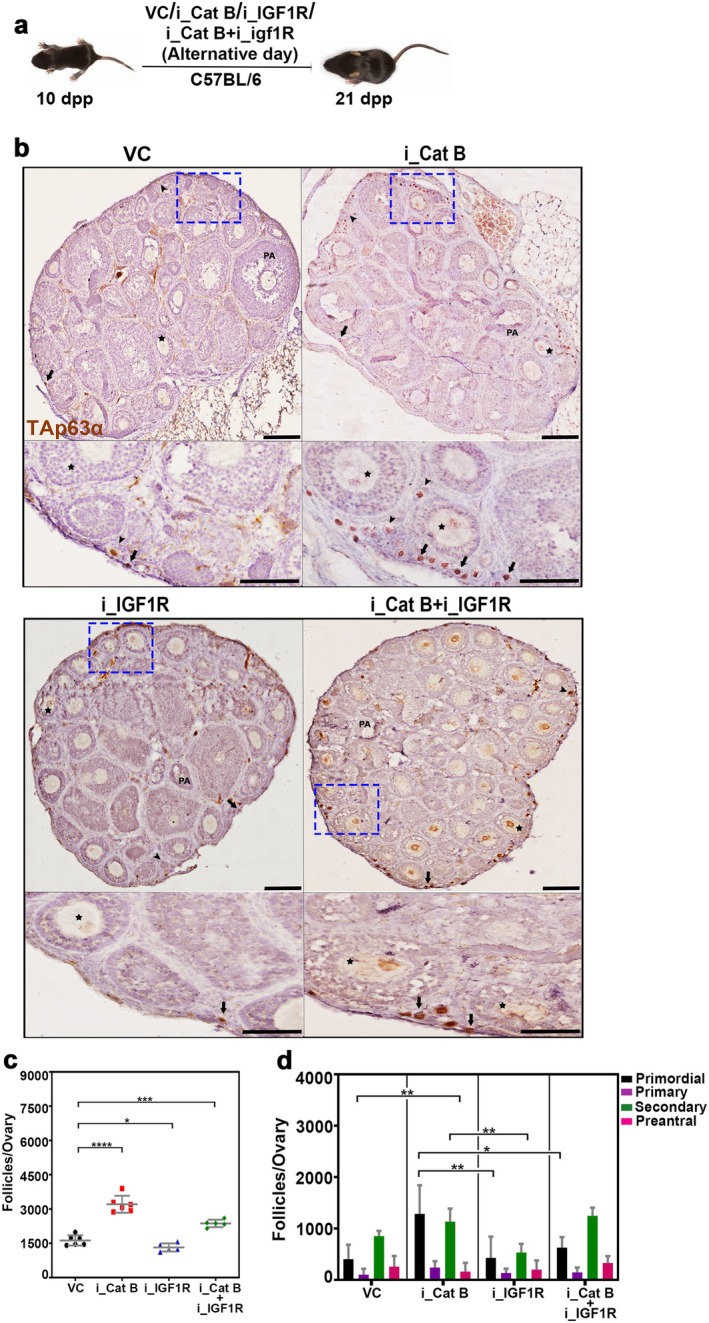
Cathepsin B protects the ovarian reserve via IGF1R. (a) Experimental regime for intraperitoneal injection of Cathepsin B and IGF1R inhibitors (*n* = 3). (b) Twenty‐one days postpartum _(dpp) ovary sections from VC‐, i_Cat B‐, i_IGF1R‐, and i_Cat B + i_IGF1R‐treated females immunostained for TAp63α with hematoxylin counterstaining. (c), Follicle counts at 21 dpp from VC‐, i_Cat B‐, i_IGF1R‐, and i_Cat B + i_IGF1R‐treated females. (d), Types of follicles at 21 dpp from VC‐, i_Cat B‐, i_IGF1R‐, and i_Cat B + i_IGF1R‐treated females. VC, vehicle control; i_Cat B, an inhibitor of Cathepsin B; i_IGF1R, an inhibitor of IGF1R; PA, preantral follicle. Arrows, arrowheads, and stars highlight the primordial, primary, and secondary follicles, respectively. The zoomed images adjacent to the ovaries are represented with blue squares. ****p ≤ 0.0001, ***p ≤ 0.0002, **p ≤ 0.02, *p ≤ 0.04, ns ≥ 0.1 unpaired *t*‐test. Error bars show mean ± SD. Nonsignificance *p* values are not represented in the figure. Scale bars for ovary sections are 100 μm.

In investigating the observed preservation of a greater number of follicles in the ovaries of mice with Cathepsin B inhibition and the concurrent elevation of IGF1R levels, we sought to understand by exploring the potential interplay between these factors. In addition, previous results suggest that IGF1R expression plays a critical role in granulosa cell survival and folliculogenesis (Baumgarten et al. 2017). To ascertain the comprehensive impact, we conducted a study where we administered a Cathepsin B inhibitor, Myricetin (37.5 mg/kg body weight), and an IGF1R inhibitor, PPP (2.5 mg/kg body weight), to 10‐day‐old female mice every alternate day with a single dose (Figure 5a). The objective was to determine whether inhibiting IGF1R in conjunction with Cathepsin B inhibition would diminish ovarian reserve. Ovaries were collected on the 21st day, fixed, sectioned, and immunostained for TAp63α to assess the outcomes of this combined inhibition strategy (Figure 5b). Analysis of follicle counts revealed that Cathepsin B‐inhibited ovaries exhibited 1.9 times as many follicles as control ovaries. In contrast, ovaries with IGF1R inhibition showed no significant increase in follicles compared to the control group. Intriguingly, mice treated with both Cathepsin B and IGF1R inhibitors displayed a 1.4‐fold reduction in follicle count compared to those treated with the Cathepsin B inhibitor alone, suggesting that the increased levels of IGF1R may indeed be responsible for the increased number of follicles in Cathepsin B‐inhibited mice ovaries (Figure 5c,d).

### 2.6 Cathepsin B Inhibition Elevates the Mitochondrial Membrane Potential via Enhanced Mitophagy

**FIGURE 6:**
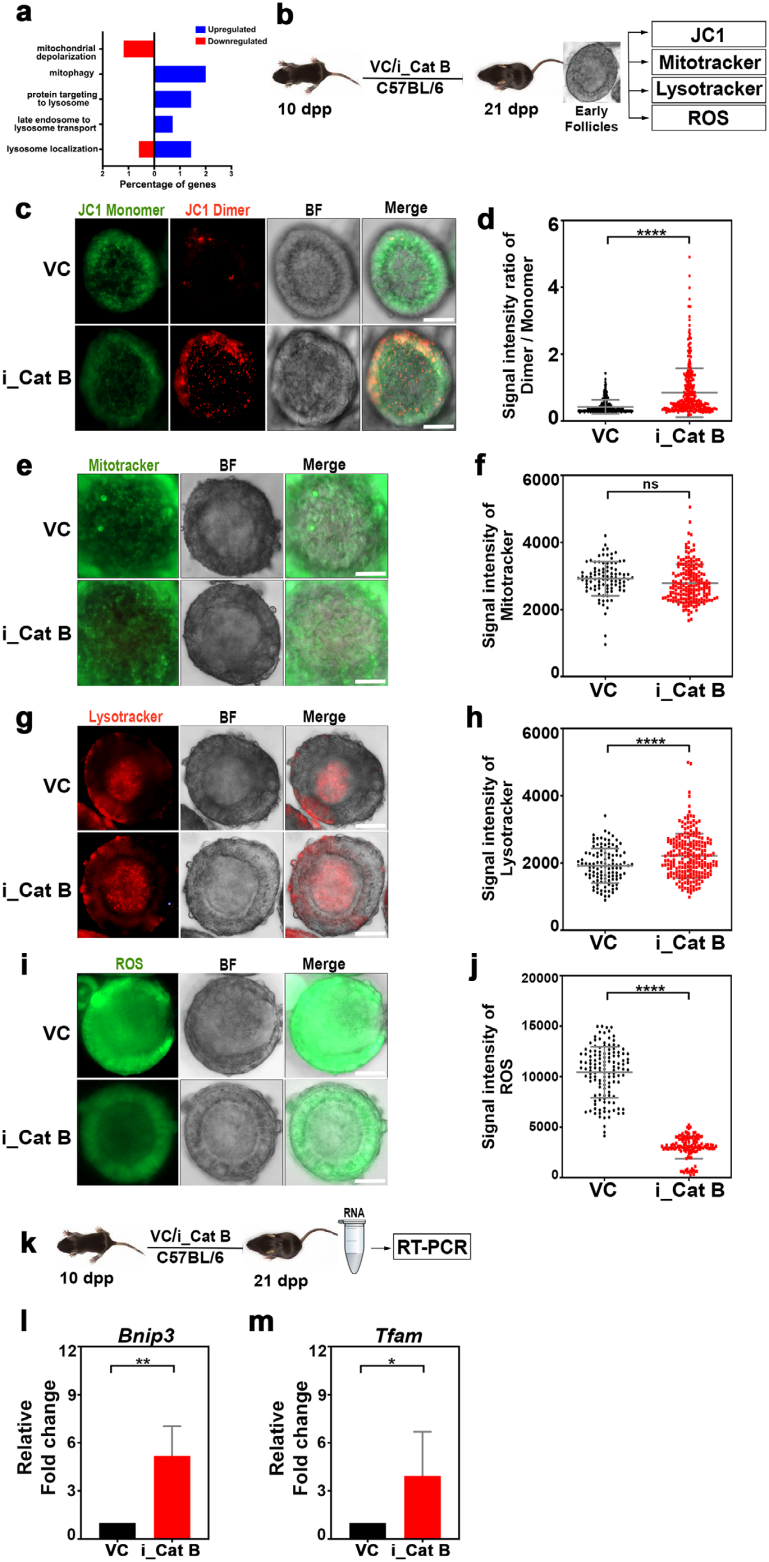
Cathepsin B inhibition regulates mitochondrial dynamics via IGF1R upregulation. (a) Relative abundance of IGF1R pathway‐related proteins in VC‐ and i_Cat B‐treated ovaries. (b) Experimental regime for intraperitoneal injection of VC‐ and i_Cat B‐treated ovaries in prepubertal mice followed by JC1, mitotracker, lysotracker, and ROS experiments (*n* = 3). (c) Early follicles from VC‐ and i_Cat B‐treated ovaries were stained for JC1 and imaged at 488 nm for JC‐1 monomers and 565 nm for dimers. (d) Quantification of the signal intensity ratio of dimer/monomer. (e) Early follicles from VC‐ and i_Cat B‐treated ovaries were stained for mitotracker green. (f) Quantification of the signal intensity of mitotracker. (g) Early follicles from VC‐ and i_Cat B‐treated ovaries were stained for lysotracker red. (h) Quantification of the signal intensity of lysotracker. (i) Early follicles from VC‐ and i_Cat B‐treated ovaries were stained for ROS. (j) Quantification of the signal intensity of ROS. (k) Experimental regime for intraperitoneal injection of VC‐ and i_Cat B‐treated ovaries followed by RT‐PCR experiments (*n* = 3). (l) and (m) Quantification of the relative fold change of *Bnip3*, *Tfam* gene expression in VC‐ and i_Cat B‐treated ovaries. VC, vehicle control; i_Cat B, an inhibitor of Cathepsin B. ****p ≤ 0.0001, **p ≤ 0.006, *p ≤ 0.04, ns ≥ 0.01 unpaired *t*‐test. Error bars show mean ± SD. Scale bars for follicles are 10 μm.

To elucidate the protective mechanism of Cathepsin B inhibition on ovarian reserve through elevated IGF1R levels, we comprehensively analyzed the proteome within IGF1R‐associated pathways in both control and Cathepsin B‐inhibited ovaries. Our findings from the proteome analysis revealed an upregulation of mitophagy and downregulation of mitochondrial depolarization in ovaries treated with Cathepsin B, as opposed to the control group (Figure 6a). In addition, previous research on mammalian cells has indicated that IGF1 signaling regulates mitochondrial turnover through mitophagy. To further investigate how enhanced mitophagy contributes to the protection of the ovarian reserve, we administered the Cathepsin B inhibitor Myricetin. For 10‐day‐old female mice, we gave a single daily dose of 37.5 mg/kg body weight, whereas 3‐month‐old mice received a double dose weekly. Ovaries were collected on the 21st day and at 4 months of age. Follicles were then isolated and classified into early and late stages based on their cumulus layers (Figure 6b, Figure S4a and Figure S5a). Early follicles were identified as those with one layer of cumulus cells, whereas late follicles had multiple layers. Following the separation, the follicles underwent live staining with JC1 (detecting mitochondrial membrane potential), Mito tracker (for mitochondrial localization), and lysotracker (for lysosomal localization) (Figure 6c,e,g, Figure S4b,d and f and Figure S5b,d and f). Analysis of JC1 in early follicles indicated an increased membrane potential in Cathepsin B treated ovarian follicles compared to controls (Figure 6d and Figure S5c). Intriguingly, the mitochondrial localization and intensity were consistent between control and treatment (Figure 6f and Figure S5e). However, lysosome activity was elevated in Cathepsin B‐treated early follicles compared to the control group, suggesting that elevated mitophagy may clear damaged mitochondria through increased lysosomal activity, contributing to the biogenesis of new mitochondria (Figure 6h and Figure S5g). Supporting this notion, we observed elevated levels of reactive oxygen species in control early follicles compared to Cathepsin B‐inhibited early follicles (Figure 6i,j and Figure S5h–i). We observed similar dynamics in late follicles. However, the fold difference is reduced compared to early follicles (Figure S4a–i). Additionally, transcription levels of *Bnip3*, a key player in mitophagy, and the essential *Tfam1* for mitochondrial biogenesis were higher in Cathepsin B treated ovaries than in controls (He et al. 2022; Picca and Lezza 2015) (Figure 6k–m). These findings collectively underscore the potential protective role of Cathepsin B inhibition in preserving ovarian reserve through complex regulation of mitophagy and mitochondrial dynamics in pre‐pubertal and post‐pubertal mice.

**FIGURE 7:**
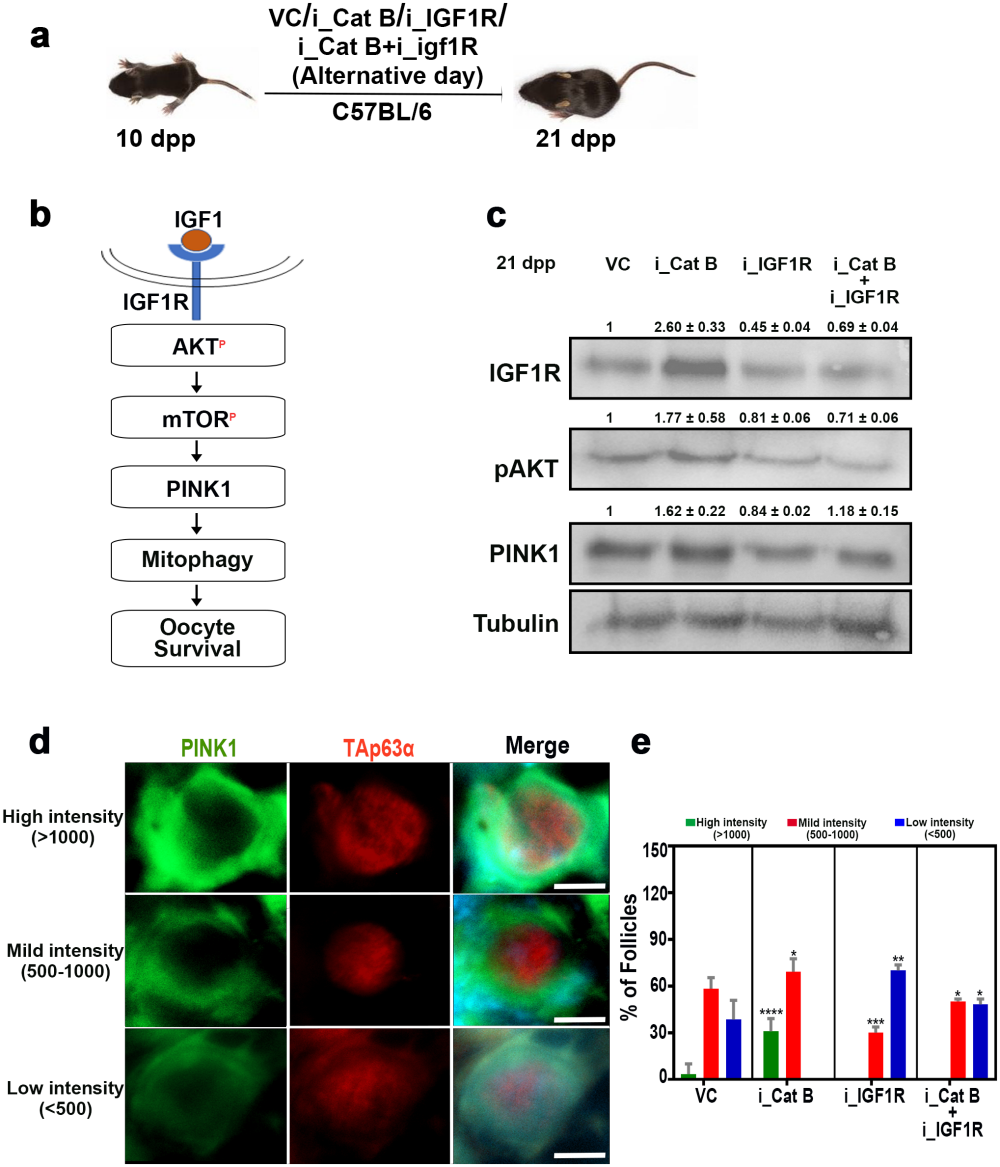
Cathepsin B protects the ovarian reserve via IGF1R‐dependent mitophagy. (a) Experimental regime for intraperitoneal injection of Cathepsin B and IGF1R inhibitors (*n* = 3). (b) Schematic representation of the insulin signaling pathway activating mitophagy in oocytes. (c) Western blot analysis of IGF1R, pAKT, PINK1, and Tubulin from 21 dpp VC‐, i_Cat B‐, i_IGF1R‐, and i_Cat B + i_IGF1R‐treated ovaries. (d) Representative image of follicles from 21 dpp ovary sections immunostained for PINK1 (green), TAp63α (red), and DNA (blue) showing different classes of PINK1 intensity. (e) Quantification of the percentage of follicles showing different classes of PINK1 intensity. VC, vehicle control; i_Cat B, an inhibitor of Cathepsin B; i_IGF1R, an inhibitor of IGF1R. ****p ≤ 0.0001, ***p ≤ 0.002, **p ≤ 0.002, *p ≤ 0.05, ns ≥ 0.2 unpaired *t*‐test. Error bars show mean ± SD. The significance *p* values shown in e represent comparisons between the control group (VC) and the treatment groups (i_Cat B or i_IGF1R or i_Cat B + i_IGF1R). Scale bars for ovary sections are 2 μm.

To investigate the mechanism underlying enhanced mitophagy following Cathepsin B inhibition and its dependence on IGF1R stability, we hypothesized that increased IGF1R signaling activates the downstream PI3K/AKT pathway. This activation modulates mitochondrial dynamics and suppresses proteases responsible for cleaving PTEN‐induced kinase 1 (PINK1). As a result, PINK1 accumulates on the outer membrane of depolarized mitochondria, marking them for degradation. Stabilized PINK1 then recruits the E3 ubiquitin ligase Parkin, leading to the ubiquitination of outer mitochondrial membrane proteins. This ubiquitination signals the selective removal of dysfunctional mitochondria through mitophagy, thereby maintaining cellular health (Figure 7b). To test this hypothesis, we administered Cathepsin B inhibitors, IGF1R inhibitors, or a combination of both to 10‐day postpartum (dpp) mice every third day until 21 dpp. Ovarian tissues were collected and analyzed for PINK1 expression across four experimental groups: control, Cathepsin B inhibition, IGF1R inhibition, and dual inhibition of both Cathepsin B and IGF1R (Figure 7a). Western blot analysis revealed a significant increase (2.6‐fold) in PINK1 levels in ovarian tissues following Cathepsin B inhibition, while IGF1R inhibition resulted in a marked reduction (0.45‐fold) in PINK1 levels. Dual inhibition of Cathepsin B and IGF1R produced PINK1 levels comparable to those observed with IGF1R inhibition alone, indicating that the mitophagy enhancement induced by Cathepsin B inhibition is dependent on IGF1R signaling (Figure 7c).

To further confirm the increase in PINK1 cytoplasmic localization, we performed immunostaining for PINK1 and TAp63α across the four experimental groups (Figure 7d). To determine the specificity of PINK1 staining, we colocalized it with Mitotracker and observed that PINK1 staining overlaps with Mitotracker, confirming its specificity (Figure S6). Further, PINK1 localization was classified into three intensity levels: (1) high intensity (> 1000), (2) mild intensity (500–1000), and (3) low intensity (< 500). Analysis of PINK1 localization revealed an increase in the proportion of oocytes exhibiting high and mild PINK1 intensity under Cathepsin B inhibition, indicating elevated mitophagic activity. In contrast, IGF1R inhibition resulted in a predominance of low‐intensity PINK1 signals, suggesting reduced mitophagy. Dual inhibition of Cathepsin B and IGF1R further exacerbated the effects observed with IGF1R inhibition alone, reinforcing the role of IGF1R in mediating PINK1 stabilization and mitophagy enhancement (Figure 7e). Collectively, these results demonstrate that Cathepsin B modulates mitophagy through IGF1R stabilization, with IGF1R playing a critical role in maintaining PINK1 expression and ensuring mitochondrial quality control.

### 2.7 Cathepsin B Inhibition Protects the Ovarian Reserve in Goat Ovaries

**FIGURE 8:**
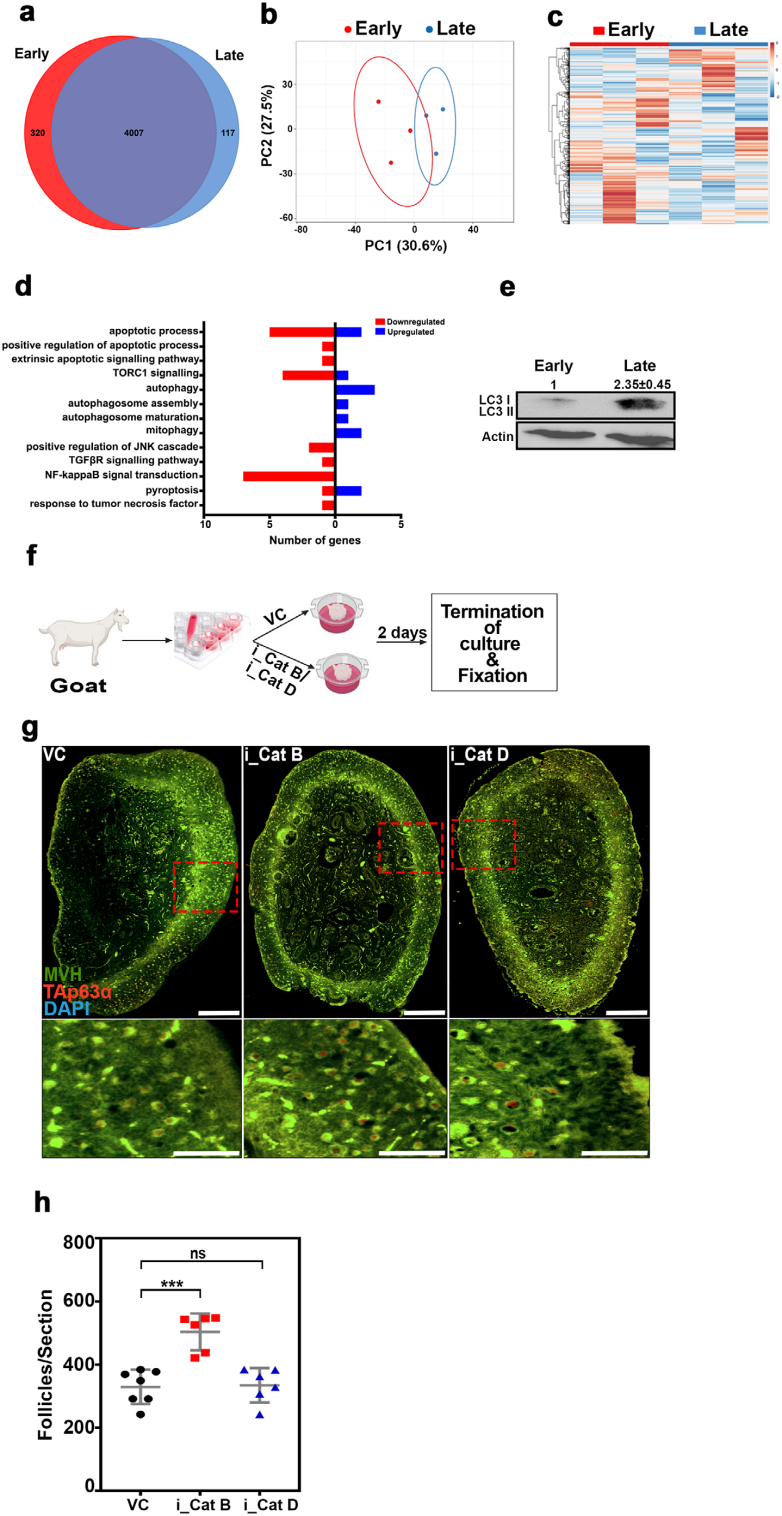
Evolutionary conservation of Cathepsin B dependent oocyte protection. (a) Venn diagram of early and late goat ovaries proteome (*n* = 3). (b) Principal component analysis (PCA) shows the difference between the early and late goat ovaries proteome profiles. (c) Relative abundance of differential proteins in early and late goat ovaries presented as a heat map. (d) Distribution of death pathways related proteins identified in early and late goat ovaries. (e) Western blot analysis of LC3 and Actin from early and late goat ovaries. (f) Experimental regime for ex vivo goat ovary culture experiments (*n* = 3). (g) VC‐, i_Cat B‐, and i_Cat D‐treated ex vivo cultured goat ovary sections immunostained for MVH (green), TAp63α (red), and DNA (blue). (h) Follicle counts at 48 h after ex vivo cultured goat ovary sections. VC, vehicle control; i_Cat B, an inhibitor of Cathepsin B; i_Cat D, an inhibitor of Cathepsin D. The zoomed images below of the ovaries are represented with red squares. ***p ≤ 0.0002, ns ≥ 0.1 unpaired *t*‐test. Error bars show mean ± SD. Nonsignificance *p* values are not represented in the figure. Scale bars for ovary sections are 200 μm.

To investigate the potential protective role of Cathepsin B inhibition on follicular reserve in higher‐order vertebrates, we conducted untargeted mass spectrometry analysis on early (1 dpp) and late (10 dpp) goat ovaries. The results revealed the identification of 4327 and 4124 proteins in early and late ovaries (*n* = 3), respectively. Notably, 320 and 117 proteins were uniquely identified in early and late ovaries, whereas 4007 were common in both samples (Figure 8a). Multivariate discriminative analysis indicated a significant separation between the two stages, with PC1 (30.6%) and PC2 (27.5%) contributing to the distinction (Figure 8b). Heat map and hierarchical clustering analysis visually confirmed distinct clusters corresponding to early and late ovaries (Figure 8c). Exploring the biological functions of the identified proteins, we observed a downregulation of apoptosis‐related genes and an upregulation of autophagy‐related genes in late ovaries compared to early ovaries (Figure 8d). Substantiating the proteomic findings, Western blot analyses validated increased autophagy levels (LC3) in late ovaries compared to early ovaries, suggesting a transition from apoptosis to autophagy during folliculogenesis in goat ovaries, similar to mice (Figure 8e). To further examine the impact of Cathepsin B inhibition, we employed an air–liquid interphase system for the in vitro culture of ovaries. Ten days postpartum goat ovaries were cultured with or without Cathepsin B and Cathepsin D inhibitors (Figure 8f). After 48 h, the ovaries were fixed, sectioned, and subjected to immunostaining for the germ cell‐specific marker, mouse vasa homolog (MVH), and the guardian of germ cells, TAp63α (Figure 8g). Analysis of follicle counts revealed a 1.5‐fold increase in follicles in ovaries treated with Cathepsin B inhibitor compared to the control group, suggesting that Cathepsin B inhibition may protect the ovarian reserve in higher‐order vertebrates (Figure 8h).

## 3 Discussion

Folliculogenesis in humans commences with an impressive count of approximately 7 million oocytes, yet only a meager 400 manage to progress to the preovulatory stage over a woman's entire lifetime. This represents less than 1% of all human oocytes, as the majority succumb to atresia during the folliculogenesis process. These phenomena are universally observed across species. Remarkably, 90%–95% of oocytes face elimination during the primordial or primary follicle stage, with the rest undergoing death at the antral follicle stage. The conventional understanding is that oocyte loss occurs in two distinct phases: first, postcoital, and second, postpartum. Postcoital oocyte loss, attributed to nest breakdown, retrotransposons, and meiotic errors, involves apoptosis and autophagy (Stringer et al. 2023). Conversely, meiotically arrested oocytes undergo maturation after birth, progressing through primordial, primary, secondary, preantral, and antral follicles. Notably, the mechanisms behind the demise of primordial and primary follicles still need to be understood. This study delves into the intricate interplay of various cell death pathways, particularly emphasizing the crucial roles of autophagy and the lysosomal cysteine protease Cathepsin B in preserving the ovarian reserve during the early postpartum stage. This stage marks the second substantial loss of follicles, offering valuable insights into the underlying processes.

### 3.1 Autophagy and Apoptosis Equally Contribute to Maintaining the Ovarian Reserve

Earlier studies in human ovaries indicate the presence of apoptosis, as observed through TUNEL, with 23.2% and 23.4% positivity in primordial and primary follicles (Depalo et al. 2003). Moreover, in mice subjected to chemotherapeutic conditions, inhibiting apoptosis preserves the ovarian reserve, implicating apoptosis in the early loss of ovarian reserve (Luan et al. 2019). However, the involvement of other death pathways remains unclear. Our initial investigation into the regulation of oocyte reserve highlights a delicate equilibrium between apoptosis and autophagy, both crucial processes in ovarian follicle development (Figure 1). Experimental inhibition of apoptosis and autophagy, particularly in early follicles, significantly amplifies the number of ovarian follicles, underscoring their pivotal roles in folliculogenesis (Figures 1 and S1). Notably, a ~2.4‐fold increase in follicle count across apoptosis and autophagy inhibitor groups suggests that these pathways contribute to ovarian reserve maintenance either independently or through interdependent mechanisms (Figure 1). However, simultaneous inhibition of both pathways proves lethal, highlighting the complexity of cell death regulation in sustaining ovarian homeostasis. Apoptosis and autophagy govern follicular atresia, and their inhibition likely disrupts normal cell turnover, resulting in enhanced follicle retention. Furthermore, all cell death pathways are interconnected through shared molecular regulators, compensatory mechanisms, and extensive crosstalk (Eisenberg‐Lerner et al. 2009). This interdependence may explain the comparable effects of inhibiting either pathway on follicle numbers. Furthermore, our results showcase a significant increase in autophagy levels during folliculogenesis or aging, establishing a correlation between autophagy and ovarian reserve. Previous studies propose that activation of the p62‐Keap1‐Nrf2 pathway plays a protective role in ovarian granulosa cells by mitigating oxidative stress and excessive autophagy, thereby reducing DEHP‐induced ovarian damage in mice. DEHP‐induced p62 accumulation competitively binds Keap1, facilitating Nrf2 nuclear translocation and establishing a positive feedback loop in antioxidant regulation (Xu et al. 2023). Moreover, pituitary‐specific p62 deficiency leads to female infertility due to impaired luteinizing hormone (LH) production, further underscoring the role of p62 in reproductive function (Li et al. 2021). Further, our untargeted proteomics analysis unravels the dynamic regulation of apoptosis and autophagy‐related genes at different stages of ovarian development, indicating a shift from apoptosis to autophagy during folliculogenesis (Figure 2a–e). This transition is further supported by Western blot analyses, reinforcing the significance of autophagy in preserving the ovarian reserve (Figure 2f–i).

### 3.2 Cathepsin B in Orchestrating Oocyte Quality and Quantity

One of the key findings of the study is the upregulation of lysosomal cysteine proteases, particularly Cathepsin B, which is similar to autophagy during folliculogenesis. Substantial evidence supports the pivotal role of Cathepsin B in programmed cell death (Chen et al. 2021). In pathological conditions, there is an increased expression and activity of Cathepsin B, leading to lysosomal membrane permeabilization and subsequent release into the cytoplasm. This release, in turn, triggers diverse programmed cell death pathways, encompassing apoptosis and autophagy (Xie et al. 2023). This study found that inhibiting Cathepsin B leads to a noteworthy increase in oocyte reserves, underscoring its crucial role in preserving ovarian reserve irrespective of age (Figure 3a–d,f–i). Genetic requirement studies indicate that Cathepsin B operates within the autophagy pathway to maintain ovarian reserve, signifying its integral involvement in ovarian reserve maintenance via autophagy (Figure S3b). Moreover, prolonged observations highlight the protective effects of Cathepsin B inhibition, demonstrating sustained preservation of primordial and primary follicles (Figure S2c). Notably, the study establishes that this protective effect is not detrimental to fertility, as evidenced by the normal reproductive outcomes in treated animals (Figure S2d–j). The study extends its scope to higher‐order vertebrates, showcasing the conservation of the Cathepsin B‐dependent mechanism in goat ovaries (Figure 8). This cross‐species validation enhances the applicability of our findings, emphasizing the evolutionary significance of this regulatory pathway in maintaining both the quality and quantity of oocytes. While our study utilized chemical inhibitors to assess the impact of Cathepsin B inhibition on ovarian reserve, leaving one fifth of the protein intact, complete knockout experiments could yield more conclusive results. Previous research has established Cathepsin B's dispensability for both male and female fertility, making it a promising therapeutic target for ovarian reserve protection (Hook et al. 2022). In addition, recent studies suggest that Cathepsin B inhibitors exhibit therapeutic potential in treating cancers and brain injuries, indicating targeting Cathepsin B may help in several pathological conditions (Kos et al. 2014; Hook et al. 2015).

### 3.3 IGF1R Links Cathepsin B to Mitochondrial Dynamics to Maintain the Quality of the Early Follicles

This study reveals a novel association between Cathepsin B and IGF1R, highlighting that inhibiting Cathepsin B leads to increased levels of IGF1R (Figure 4e–h). In vitro experiments further illustrate that IGF1R serves as a substrate for Cathepsin B (Figure 4i–n). Exploring the downstream effects, our study elucidates that elevated IGF1R levels impact mitochondrial membrane potential through the transcriptional regulation of genes associated with mitochondrial biogenesis and mitophagy, providing a mechanistic understanding of how Cathepsin B influences oocyte quality (Figures 6c,d,k–m and S5b. c). Our findings align with previous research indicating that IGF1 signaling governs mitochondrial dynamics and turnover in cancer cells through the NRF2‐BNIP3 pathway (Riis et al. 2020; Lyons et al. 2017). These results suggest that the protective effects of inhibiting Cathepsin B on ovarian reserve may be attributed to enhanced mitophagy, contributing to the preservation of high‐quality oocytes. In addition, PINK1, a mitochondrial serine/threonine‐protein kinase, is integral to mitochondrial quality control. It identifies damaged mitochondria and targets them for degradation through mitophagy. Our observations indicate that inhibiting Cathepsin B leads to an accumulation of PINK1, while inhibiting IGF1R results in decreased PINK1 levels. Notably, dual inhibition of both Cathepsin B and IGF1R produces PINK1 levels similar to those observed with IGF1R inhibition alone, suggesting that the enhancement of mitophagy induced by Cathepsin B depends on IGF1R signaling (Figure 7). Then the question is, what role does Cathepsin B play in regulating ovarian reserve? Our findings indicate that elevated oxidative stress in the follicular microenvironment may harm the mitochondria of oocytes (Figure 6i, j and S5h, i). Consequently, these oocytes with damaged mitochondria might experience cell death by suppressing mitophagy through the regulation of IGF1R turnover. A crucial observation is that even though all oocytes may encounter varying levels of oxidative stress, only a select few undergo cell death. This inconsistency could be attributed to differing Cathepsin B and IGF1R levels in the follicular environment (Figure S7a–f). Our study unveiled variations in the localization of Cathepsin B and IGF1R in different follicles, suggesting that follicles undergoing cell death might exhibit higher Cathepsin B levels. This increase in Cathepsin B might potentially decrease IGF1R levels, subsequently reducing mitophagy and leading to follicular death (Figure S7a–f). Further research is required to substantiate this hypothesis. In conclusion, this study significantly advances our comprehension of the molecular intricacies involved in maintaining oocyte reserves. The roles identified for autophagy, Cathepsin B, and IGF1R provide valuable insights that may shape the development of innovative fertility treatments and interventions.

### 3.4 Limitations

While the long‐term effects of Cathepsin B inhibition, including potential lysosomal dysfunction or off‐target interactions, have not been explicitly investigated in this study, the inhibitors utilized have been previously approved for the treatment of cancer, neurological, cardiovascular, and other diseases. Their established clinical use suggests a generally favorable safety profile. However, given the differences in physiological contexts and therapeutic applications, further research is required to rigorously evaluate their safety, efficacy, and potential unintended consequences in the context of fertility treatment.

## 4 Materials and Methods

### 4.1 Animal Experiments

The mice were maintained on a 12‐h light–dark cycle with food and water provided ad libitum in accordance with the guidelines set forth by the National Institute of Animal Biotechnology Ethics Committee. C57BL/6 mice were utilized throughout the experiments, except for the evaluation of oocyte and embryo quality, for which FVB mice were employed. In experiments concerning death pathways, 5 postnatal days (dpp) (*n* = 4 to 7 per inhibitor) were divided into two groups: vehicle control (VC) and inhibitors of apoptosis (i_Apo) [MCE‐ HY‐16658B (Z‐VAD‐FMK) 5 mg/kg body wt.], autophagy (i_Aut) [TCI‐M2518 (3‐MA) 50 mg/kg body wt.], pyroptosis (i_Pyr) [Sigma‐PHR1690 (Disulfiram) 10 mg/kg body wt.], and necroptosis (i_Nec) [Sigma‐5303890001 (GSK' 872) 5 mg/kg body wt.]. The inhibitors were administered intraperitoneally every other day from 5 to 10 dpp at specified doses. Ovaries were harvested at 10 dpp to maximize the observation of inhibitor effects during the period of higher follicular numbers. For Cathepsin experiments in prepubertal mice, 10 dpp pups (*n* = 3–8 per group) were divided into VC, i_Cat B [SRL‐15358, 37.5 mg/kg body wt.], and i_Cat D [Sigma‐77170, 20 mg/kg body wt.] groups. Based on previous studies and lethality assessments, the pups received intraperitoneal injections of Cathepsin B (i_Cat B) or Cathepsin D (i_Cat D) inhibitors. Ovaries were collected on 21 dpp, with a subset of i_Cat B pups aged 6 months to evaluate long‐term effects. For Cathepsin experiments in postpubertal mice, 3‐month‐old pups (*n* = 2–3 per group) were divided into VC and i_Cat B [SRL‐15358, 37.5 mg/kg body wt.] groups. The inhibitor was administered intraperitoneally weekly twice at specified doses and Ovaries were collected at 4 months. To investigate the interaction between IGF1R and Cathepsin, 10 dpp pups (*n* = 5–6 per inhibitor group) were divided into VC, i_IGF1R [Selleck‐S7668, 2.5 mg/kg body wt.], i_Cat B, and i_IGF1R + i_Cat B groups. Intraperitoneal injections of the respective inhibitors were administered every other day from 10 dpp to 20 dpp, and ovaries were collected at 21 dpp. Additional studies involved the administration of i_Cat B to 10 dpp pups for JC1, mitotracker, lysotracker, and ROS assessments, with ovaries collected on 21 dpp. Endocrine profiling and fertility tests were conducted by injecting i_Cat B into 10 dpp pups, whereas oocyte and embryo assessments were performed on 10 dpp FVB mice pups treated with i_Cat B until 20 dpp, followed by sacrifice on 45 dpp.

### 4.2 Assessment of Mitochondrial Distribution, Membrane Potential, ROS, and Lysosome Activity

The ovaries were delicately trimmed and sliced using a fine 31‐gauge needle immersed in M2 media (Sigma‐M7167). Subsequently, follicles were carefully isolated under microscopic observation and thoroughly rinsed in media. These follicles were then categorized based on the number of cumulus cell layers they possessed: those with a single layer were denoted early follicles, while those with two or more layers were classified as late follicles. For the assessment of mitochondrial distribution and membrane potential, the follicles were treated with either 2 μM of JC‐1 (ThermoFisher—M34152) or 40 μM of Mito Tracker Green FM (ThermoFisher—M7514) in M2 media at a temperature of 37°C for 30 min. Following this incubation period, the follicles underwent thorough washing with M2 media and subsequent rinsing with 1× PBS before being transferred to a dish for immediate microscopic imaging. Excitation at 488 nm was utilized for mitotracker Green, whereas JC1 fluorescence was captured at 488 nm and 555 nm. The fluorescence intensity ratio at 555 nm to that at 488 nm was computed to determine the membrane potential. To evaluate lysosomal activity, follicles were exposed to 0.2 μM lysotracker (ThermoFisher—L7528) for 5 min, followed by a series of rinses: three with M2 media and three with 1× PBS. Microscopic images were quickly captured with excitation at 555 nm. For the quantification of reactive oxygen species (ROS) levels, follicles were treated with 10 μM of 2',7' dichlorodihydrofluorescein diacetate (H2DCFDA) (Invitrogen‐D399) for 5 min and then subjected to similar washing steps before immediate microscopic imaging.

### 4.3 RNA Isolation and mRNA Expression

Upon sacrificing, ovaries were collected in 1 mL of TRIzol (Takara—9108). Then, they were chopped finely, followed by vortexing and centrifugation at 10,000 rpm for 2 min at 4°C. The supernatant was collected, and RNA was isolated using the phenol‐chloroform method. RNA concentration was measured, and cDNA was synthesized according to manufacturer instructions (Takara—6110a). One hundred nanograms of cDNA was used to check the mRNA expression of BNIP3, TFAM, Actin, and GAPDH (primers in Table 1) in ovaries by using TB Green syber mix (Takara—RR820A) as per manufacturer instructions. The relative expression of the PCR product was quantified using the (−ΔΔ^C^_t_) method following Livak's approach. Statistical significance was established at 0.05% (*p* < 0.05).

**TABLE 1:** Primers used in the qPCR assays.

Gene and ID	Forward primer	Reverse primer	Annealing temperature (°C)	Product size (bp)
BNIP3 (MGI:109326)	GCTCCCAGACACCACAAGAT	TGAGAGTAGCTGTGCGCTTC	62	222
TFAM (MGI:107810)	TAGTGTGGCAGTCCATAGGC	GCTTTTAGCACGCTCCACAT	62	115
GAPDH (MGI:95640)	TTGTCAAGCTCATTTCCTGGTATG	GCCATGTAGGCCATGAGGTC	62	76
Actin (MGI:87904)	ATATCGCTGCGCTGGTCGTC	ATAGGAGTCCTTCTGACCCATT	62	149

### 4.4 Endocrine Profiling

The serum samples were processed for LH (KLM0573), FSH (K02‐0257), and AMH (KLM1096) profiling according to the manufacturer's protocol.

### 4.5 Oocyte and Embryo Culture

Ovaries were trimmed and punctured with a 24‐gauge needle in M2 media (Sigma‐M7167). Under microscopic observation, oocytes were delicately retrieved from the media and thoroughly cleansed through multiple washes. Subsequently, these oocytes were allowed to mature within the same M2 media within the controlled environment of a CO_2_ incubator set to 90%–95% humidity and 37°C. After 16 h of incubation, the oocytes were examined for signs of maturation (polar body extrusion and DNA staining). To collect embryos, animals underwent superovulation through injections of PMSG and HCG. Once primed, the ovaries and the oviducts from plugged females were carefully dissected. The oviducts underwent trimming, and swollen ampullas were gently punctured using a 24‐gauge needle within M2 Media. Embryos, still encased in cumulus complexes, were washed twice with M2 media and once with Embryomax HTF media (Sigma‐ MR‐070‐D). Following this, the embryos were cultured using the same Embryomax HTF media within a 4‐well IVF dish (Thermo‐144444), all within the controlled conditions of a CO_2_ incubator set to 90%–95% humidity and 37°C. After 4 days, the embryos were microscopically observed for signs of maturation.

### 4.6 Fertility Studies

Individual adult female mice, treated and untreated, were housed separately with untreated males to evaluate fertility. If a female failed to conceive within a month, the male partner was replaced. The females underwent plug‐checking for a week or until successful mating with a new male, confirmed by the presence of a mating plug.

### 4.7 Goat Ovary Culture

Ovary samples from the Osmanabadi goat breed obtained 1 day after birth were collected from the slaughterhouse and immediately immersed in a warm saline solution (32°C–37°C) fortified with penicillin–streptomycin antibiotics. Within an hour of collection, the samples were transported to the laboratory for further processing using an embryo carrier. Upon arrival, the ovaries were carefully trimmed and placed on a filter strip (782810). This filter was then floated on a nutrient medium consisting of TCM199 (Sigma‐M2154) supplemented with 10% goat follicular fluid, 1× penicillin–streptomycin (Sigma‐P4333), and 3% fetal calf serum. The culture plates (782891) containing the samples were then placed in a controlled environment containing a CO_2_ incubator set at 37°C with 90%–95% humidity. After 2 h of culture, either 50 μM of i_Cat B or 1 μM of i_Cat D, along with their respective controls, were introduced into the culture. The culture was allowed to continue for a total of 48 h. Upon completion of the culture period, the ovaries were fixed in 10% formalin and underwent further processing for sectioning. All experiments were conducted with three independent biological replicates to ensure the reliability and consistency of the results.

### 4.8 In Vitro Cathepsin B Reaction

For in vitro reaction, 1 μg of purified IGF1R (R&D systems‐305‐GR) was incubated with assay buffer 30 μL (25 mM MES [pH 5]) in the presence or absence of purified Cat B (R&D systems‐953‐CY [500 ng]). After 2 h incubation at 37°C, the reaction was kept at −20°C to quench the reaction.

### 4.9 MS Sample Preparation

For whole‐cell untargeted proteomics of ovarian samples, initially, ovaries were lysed using a solution containing 1% Sodium Deoxycholate (SDC) prepared in 50 mM Ammonium Bicarbonate Buffer (AMBIC). Following this, the protein concentration in the lysate was determined using the Bradford assay, and equal amounts of protein from each sample were selected for subsequent processing. Samples subjected to in vitro reactions were directly processed after the reaction. The process began with treating the samples with 200 mM Dithiothreitol (DTT) to reduce disulfide bonds, which was carried out at 57°C for 1 h. Subsequently, alkylation was performed using 200 mM Iodoacetamide (IAA) at room temperature in the dark for 1 h. The samples were then digested using a combination of trypsin and LysC enzymes at 37°C for 16 h. To remove sodium deoxycholate (SDC) from the trypsin‐digested samples, 20% formic acid was added to precipitate it. The resulting mixture was then passed through a 10 kDa filter to eliminate undigested proteins. Further purification was achieved by desalting the samples using a C18 spin column following the manufacturer's instructions. The purified samples were subsequently vacuum‐dried. Further, the dried samples were reconstituted in 0.3% formic acid. Finally, 1 μg of the peptide sample was injected into the mass spectrometer for analysis. All experiments were done with two to three independent biological replicates.

### 4.10 Mass Spectroscopy Data Acquisition

The proteome was analyzed by using the UltiMate 3000 RSLCnano system coupled with the high‐resolution Q Exactive HF mass spectrometer (ThermoFisher Scientific). In brief, the full MS scans were carried out by using a resolution value of 60,000, AGC target value of 1 × 10^6^ and acquisition range of 375–1600 *m/z* with a maximum injection time of 60 ms. The top 25 precursors were selected for the fragmentation. The MS2 acquisition was performed through a resolution of 15,000, AGC target value of 1 × 10^5^, a maximum injection time of 100 ms, an isolation window of 1.3 *m/z*, and a fixed first mass at 100 *m/z*. A nonlinear gradient (flow rate of 0.300 μL/min for 180 min) of solvents using 5% of 80% acetonitrile/0.1% formic acid as solvent B and 95% of 0.1% formic acid as solvent A was preferred for eluting the peptides.

### 4.11 Mass Spectroscopy Data Analysis

The peptides and proteins were identified by using the software Proteome discoverer v2.5 (ThermoFisher Scientific, San José, CA, USA) with UniProtKB/Swiss‐Prot: P08069.1, UniProtKB Mice (UP000000589) and Goat (UP000291000) database.

### 4.12 Parameters for Peptide and Protein Search

We selected the dynamic and static modifications of oxidized methionine residues and carbamidomethylation of cysteine residues as our prime search parameters. In addition, we considered the following values of 2, 144, and 6 for most missed cleavage, maximum, and minimum peptide length, respectively. Proteins and peptides with < 1% and < 5% FDR confidence were filtered. The identification of proteins and peptides were based on the 10 ppm and 0.02 Da fragment and precursor mass tolerances. The “Minora Feature Detector,” “Precursor Ions Quantifier,” and “Feature Mapper” nodes workflow were used for the Label‐free quantification (based on peptide signals) to identify proteins or peptides (Matthiesen and Jensen 2008).

### 4.13 Bioinformatics and Statistical Analysis

Following the label‐free quantification, protein intensities (abundance values) were normalized by log2 transformation. The intervariation and intravariation between different samples were determined by using the Venn diagram and PCA, respectively. Heat map and PCA graphs were generated by using Clutvis (http://biit.cs.ut.ee/clustvis/). Gene ontology (GO) comparative analysis was performed with Funrich v3.1.3 and ShinyGO v0.741 software (Carnielli et al. 2015; Schmidt et al. 2014).

### 4.14 Cell Culture

Human lung adenocarcinoma cell line H1299 purchased from ATCC (ATCC—CRL‐5803) was grown in RPMI‐1640 (Gibco—11875085), 10% FBS (Gibco—10270106) and 1× penicillin–streptomycin (Sigma—P4333) at 37°C, and 5% CO_2_. For Cat B overexpression, the cells were transfected with hCathepsin B (add gene‐11249) plasmid using Lipofectamine 3000 transfection Reagent (Thermo‐ L3000008) following the manufacturer's protocol. The cells were harvested after 24 h of transfection. To inhibit Cathepsin B, cells were cultured with or without i_Cat B (50 μM) for 4 h and then harvested. Harvested cells were then centrifuged at 15,000 rpm at 4°C for 10 min to collect the pellets. The pellets were resuspended in lysis buffer (25 mM Tris–HCL, pH‐8.0; 250 mM NaCl; 1% Triton‐X‐100; 1% SDS; 2 mM MgCl2; PMSF; Protease inhibitor) followed by sonication and centrifugation at 12,000 rpm at 4°C for 10 min. Supernatants were then boiled at 100°C for 5 min with 1× Laemmli buffer and loaded in SDS‐PAGE to check the protein of interest.

### 4.15 Immunohistochemistry and Immunofluorescence

The ovaries were first fixed in 10% formalin for 2–4 days. Fixation was followed by a gradient treatment of 10%, 20%, and 30% sucrose up to 3 days depending on the size of the ovary. Then 5 μm thin cryosections were obtained by the help of Cryostar NX50 and placed on glass slides with positive surface coating. The slides were then deparaffinized for further staining processes. Next the slides were treated at 95°C for 60 min in antigen retrieval buffer comprising 10 mM Sodium Citrate and 0.05% Tween 20 for antigen retrieval necessary in further immunofluorescence staining. After cooling to room temperature under running water, the tissue sections on slides underwent two times dH_2_O‐based washing for 5 min each followed by rinsing with 1× TBST‐0.1% Tween 20 for 1 min. The tissue sections were then blocked with M.O.M (BMK‐2202) for 1 h at room temperature, followed by two rounds of blocking with ADB for 15 min each. The sections were incubated overnight in primary antibodies (Rabbit anti‐TAp63α [BS1279, 1:500], Mouse anti‐MVH [ab27591, 1:500], Rabbit anti‐PINK1 [D8G3, 1:250], Mouse anti‐TAp63α [CM 163B, 1:500]). Following incubation, slide containing sections were washed two times with 1× TBST‐0.1% Tween 20 in 10 min intervals. Then slides were blocked with ADB twice for 15 min each. Afterward, the slides were incubated with corresponding secondary antibodies (Goat anti‐rabbit 555 [A32732, 1:2000], Goat anti‐mouse 488 [A32723, 1:2000], Goat anti‐rabbit 488 [A32731, 1:2000], Goat anti‐mouse 555 [A32727, 1:2000]) at 37°C for 1 h in the dark. Later, the slides were washed thrice with 1× TBST‐0.1% Tween 20 for 5 min each and stained with 0.01% DAPI for 2 min. The slides were then air‐dried after a minute of dH_2_O washing. For DAB staining, after blocking with ADB, the slides were incubated with secondary antibody Goat anti‐rabbit biotin (B‐2770, 1:1000) at 37°C for 1 h in the dark.

This was followed by three times washing with 1× TBST‐0.1% Tween 20 at 5 min intervals. Subsequently, slides were processed according to the manufacturers protocol, that is, the VECTASTAIN ABC‐HRP kit (PK‐6100) and DAB substrate kit (SK‐4100). The slides were then dried and mounted with DPX, and images were captured under the microscope.

### 4.16 Imaging and Quantification

Immunostained slide images were captured by the help of Zeiss Axio scope VII microscope with 10×, 20× Plan Apochromat 0.45 NA, or 63× Plan Apochromat 1.4 NA objectives and EXFO X‐Cite metal halide light source with a Hamamatsu ORCA‐ER CCD camera. The acquired images were processed by the Zen software. In the ovarian samples, we examined every fifth section for different types of follicles: primordial, primary, secondary, preantral, and antral. A single layer of squamous or cuboidal epithelial cell containing follicles was considered primordial or primary follicles, respectively. In a similar fashion, follicles surrounded by two or more layers of cells were considered secondary follicles. Follicles with early antrum formation were counted as preantral follicles, and completely formed antrum containing follicles were considered antral follicles. All quantitative analyses were executed by two observers, where the second observer was blindfolded in the examined group.

### 4.17 Western Blotting

The ovaries were minced and lysed in a lysis buffer containing 25 mM Tris–HCL (pH 7.5), 150 mM NaCl, and 1% Triton‐X‐100, supplemented with PMSF and Protease inhibitor. Next, the mixture was homogenized, sonicated, and centrifuged at 12,000 rpm for 10 min at 4°C. The supernatant was collected carefully and stored at −80°C for further analysis. The protein concentration was determined using the Bradford assay kit (Takara). For the vehicle control and inhibitor‐treated or 5, 10, and 20 dpp ovarian samples, equal amounts of protein were loaded. The proteins were separated by SDS‐PAGE and then transferred onto a nitrocellulose membrane. The membrane was then blocked with 5% skimmed milk for 1 h at room temperature, followed by overnight incubation at 4°C with primary antibodies. The primary antibodies used were as follows: Mouse anti‐γH2A.X (05‐63‐I, 1:1000), Rabbit anti‐cleaved Caspase 3 (D175, 1:1000), Rabbit anti‐LC3 (NB100‐2220, 1:1000), Rabbit anti‐P62 (5114T, 1:1000), Rabbit anti‐TAp63α (BS1279, 1:1000), Rabbit anti‐MVH (ab13840, 1:1000), Mouse anti‐Cathepsin B (C6243, 1:500), Rabbit anti‐Cathepsin D (E7Z4L, 1:1000), Mouse anti‐ATG7 (MAB6608, 1:1000), Mouse anti‐IGF1 (sm 1.2, 1:1000), Rabbit anti‐IGF1R (9750, 1:1000), Rabbit antiphospho‐IGF1R (3021, 1:1000), Rabbit antiphospho‐AKT (4060, 1:1000), Rabbit antiphospho‐mTOR (5536, 1:1000), Mouse anti‐PINK1 (N4/15, 1:1000), Mouse anti‐Tubulin (SC 53646, 1:1000), and Mouse anti‐β‐Actin (SC‐47778, 1:1000). After the overnight incubation, the blots were washed thrice at an interval of 5 min with 1× TBST consisting of 0.3% Tween 20 and then incubated with the corresponding secondary antibodies labeled with HRP (Goat anti‐Mouse IgG HRP, 31430; Goat anti‐Rabbit IgG, 31460) for 2 h at room temperature. After three rounds of washing with 1× TBST containing 0.3% Tween 20 and a final wash with 1× TBS, the blots were developed using chemiluminescence. The Western blot signals were quantified using ImageJ software.

## Author Contributions

A.M. and H.B.D.P.R. conceived the study and designed the experiments. A.M., A.K., A.K., K.K.P., L.K.S., R.B., P.B., M.A. and H.B.D.P.R. performed the experiments and analyzed the data. A.M., and H.B.D.P.R. wrote the manuscript with inputs and edits from all authors.

## Conflicts of Interest

The authors declare no conflicts of interest.

## Supporting information


**Figure S1.** (a–c) Quantification of primordial, primary, and secondary follicles at 10 dpp from VC, i_Apo, i_Aut, i_Pyr, and i_Nec ‐treated mice ovaries. VC, vehicle control; i_Apo, an inhibitor of apoptosis; i_Aut, an inhibitor of autophagy; i_Pyr, an inhibitor of pyroptosis; i_Nec, an inhibitor of necrosis. ****p ≤ 0.0001, ***p ≤ 0.001, **p ≤ 0.004, *p ≤ 0.04, ns ≥ 0.1 unpaired *t*‐test. Error bars show mean ± SD.
**Figure S2.** (a) Experimental regime for i_Cat B intraperitoneal injection with different doses and in aged mice ovaries (*n* = 3–5). (b) Follicle counts at 21 dpp with different doses. (c) Follicle counts at 6 months of 11‐dose treatment of VC and i_Cat B mice ovaries, respectively. (d) Experimental regime for i_Cat B intraperitoneal injection followed by endocrine profiling, oocyte, embryo quality assessment, and fertility test experiments. (e) Quantification of LH, FSH, and AMH in the serum from VC‐ and i_Cat B‐treated females. (f) Bright field oocyte maturation images. (g) Quantification of in vitro oocyte maturation from VC and i_Cat B‐treated females. (h) Bright field embryo development images. (i) Quantification of in vitro embryo development from VA and i_Cat B‐treated females. (j) Fertility performance of VC and i_Cat B‐treated females. VC, vehicle control; i_Cat B, an inhibitor of Cathepsin B. ****p ≤ 0.0001, **p ≤ 0.001, *p ≤ 0.02, ns ≥ 0.5 unpaired *t*‐test. Error bars show mean ± SD. Scale bars are 20 μm.
**Figure S3.** (a) Experimental regime for i_Cat B intraperitoneal injection with different death pathways inhibitors (*n* = 3–4). (b) Follicle counts at 10 dpp from VC and i_Cat B alone or along with i_Apo‐, i_Aut‐, i_Pyr‐, and i_Nec‐treated females. VC, vehicle control; i_Cat B, an inhibitor of Cathepsin B; i_Apo, an inhibitor of apoptosis; i_Aut, an inhibitor of autophagy; i_Pyr, an inhibitor of pyroptosis; i_Nec, an inhibitor of necrosis. **p ≤ 0.006, *p ≤ 0.01, ns ≥ 0.5 unpaired *t*‐test. Error bars show mean ± SD. Nonsignificant *p* values are not represented in the figure.
**Figure S4.** (a) Experimental regime for intraperitoneal injection of VC and i_Cat B ‐treated ovaries in prepubertal mice followed by JC1, mitotracker green, lysotracker red, and ROS experiments (*n* = 3). (b) Late follicles from VC and i_Cat B‐treated ovaries were stained for JC1 and imaged at 488 nm for JC‐1 monomers and 565 nm for dimers. (c) Quantification of the signal intensity ratio of Dimer/Monomer. (d) Late follicles from VC and i_Cat B‐treated ovaries were stained for mitotracker. (e) Quantification of the signal intensity of mitotracker. (f) Late follicles from VC and i_Cat B ‐treated ovaries were stained for lysotracker. (g) Quantification of the signal intensity of lysotracker. (h) Late follicles from VC‐ and i_Cat B‐treated ovaries were stained for ROS. (i) Quantification of the signal intensity of ROS. VC, vehicle control; i_Cat B, an inhibitor of Cathepsin B. ****p ≤ 0.0001, **p ≤ 0.07, ns ≥ 0.1 unpaired *t*‐test. Error bars show mean ± SD. Scale bars for follicles are 10 μm.
**Figure S5.** (a) Experimental regime for intraperitoneal injection of VC and i_Cat B‐treated ovaries in postpubertal mice followed by JC1, mitotracker green, lysotracker red, and ROS experiments (*n* = 3). (b) Late follicles from VC‐ and i_Cat B‐treated ovaries were stained for JC1 and imaged at 488 nm for JC‐1 monomers and 565 nm for dimers. (c) Quantification of the signal intensity ratio of Dimer/Monomer. (d) Late follicles from VC‐ and i_Cat B‐treated ovaries were stained for mitotracker. (e) Quantification of the signal intensity of mitotracker. (f) Late follicles from VC‐ and i_Cat B‐treated ovaries were stained for lysotracker. (g) Quantification of the signal intensity of lysotracker. (h) Late follicles from VC‐ and i_Cat B‐treated ovaries were stained for ROS. (i) Quantification of the signal intensity of ROS. VC, vehicle control; i_Cat B, an inhibitor of Cathepsin B. ****p ≤ 0.0001, **p ≤ 0.005, *p ≤ 0.04, ns ≥ 0.1 unpaired *t*‐test. Error bars show mean ± SD. Scale bars for follicles are 10 μm.
**Figure S6.** (a) Representative images of follicles from 21 dpp mice ovary immunostained for mitotracker (Green), PINK1 (Red), and DNA (blue) or PINK1 (Green), TAp63α (Red) and DNA (blue). The zoomed images of oocyte on the follicle sections are represented with white squares. Scale bars for follicles are 10 μm.
**Figure S7.** (a) Representative images of early follicles from 10 dpp mice ovary immunostained for Cathepsin B (red), IGF1R (green), and DNA (blue). (b) and (c) Quantification of the signal intensity of Cathepsin B and IGF1R of early follicles at high and low intensity of Cathepsin B, respectively. (d) Representative images of late follicles from 10 dpp mice ovary immunostained for Cathepsin B (Red), IGF1R (green), and DNA (blue). (e) and (f) Quantification of the signal intensity of Cathepsin B and IGF1R of late follicles at high and low intensity of Cathepsin B, respectively. (g) Experimental regime for intraperitoneal injection of Cathepsin B inhibitor (*n* = 3). (h) Representative images of early follicles of 21 dpp ovary sections from VC‐ and i_Cat B‐treated females immunostained for Cathepsin B (Red), IGF1R (green), and DNA (blue). (i) and (j) Quantification of the signal intensity of Cathepsin B and IGF1R of early follicles, respectively. (k) Representative images of late follicles of 21 dpp ovary sections from VC and i_Cat B‐treated females immunostained for Cathepsin B (Red), IGF1R (green), and DNA (blue). (l) and (m) Quantification of the signal intensity of Cathepsin B and IGF1R of late follicles, respectively. ****p ≤ 0.0001, **p ≤ 0.002, *p ≤ 0.04, unpaired *t*‐test. Error bars show mean ± SD. Scale bars for early and late follicles are 2 μm and 10 μm, respectively.
**Figure S8.** (a–h) Whole western blots.

## Data Availability

The data that support the findings of this study are available from the corresponding author upon request.
