# Supplementary figures and images for: Cathepsin B Regulates Ovarian Reserve Quality and Quantity via Mitophagy by Modulating IGF1R Turnover
Source: Aging Cell. 2025 Apr 28;24(7):e70066. doi: 10.1111/acel.70066 (PMC12266751; doi:10.1111/acel.70066)

FigS1:


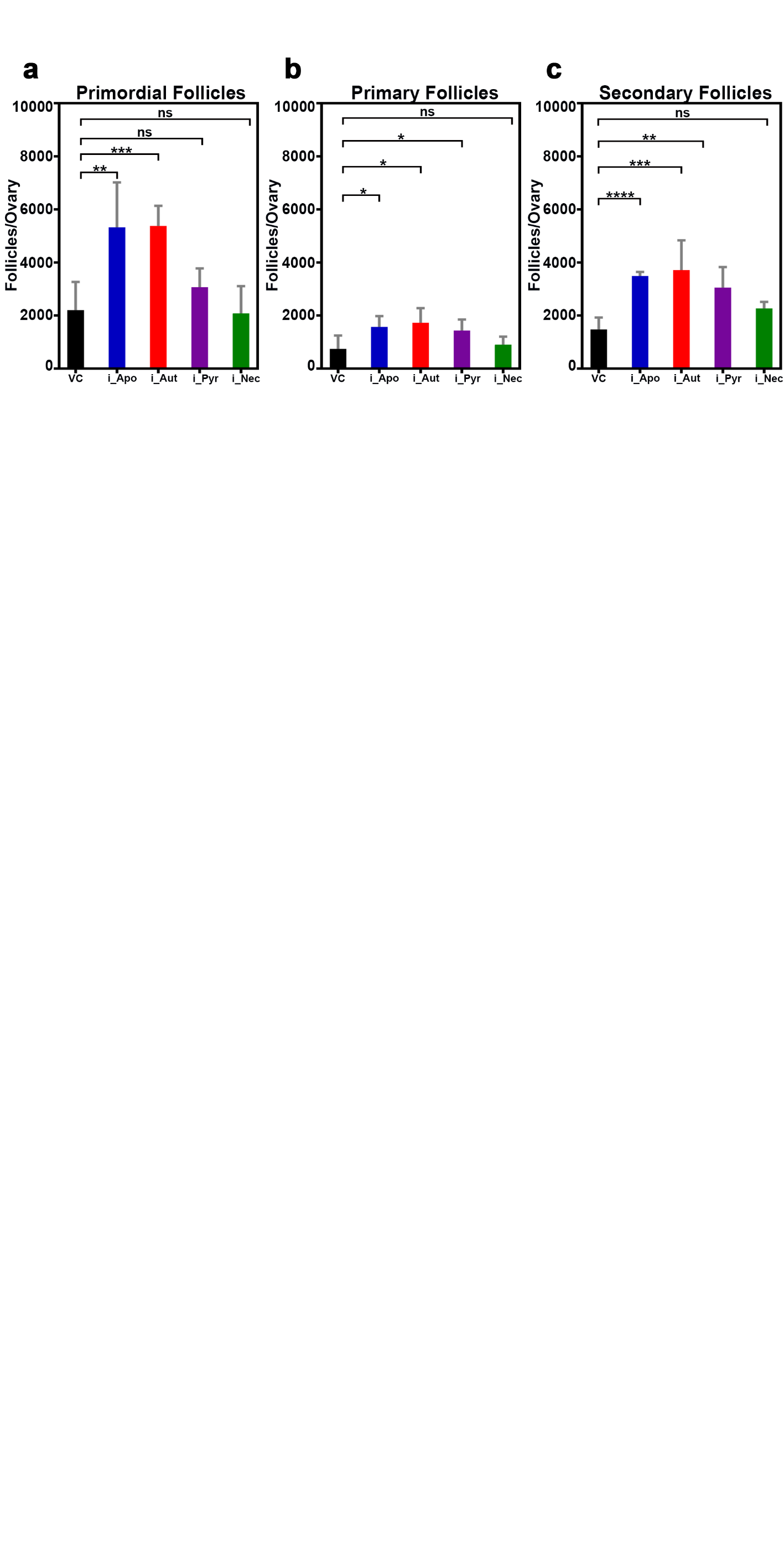


FigS2:


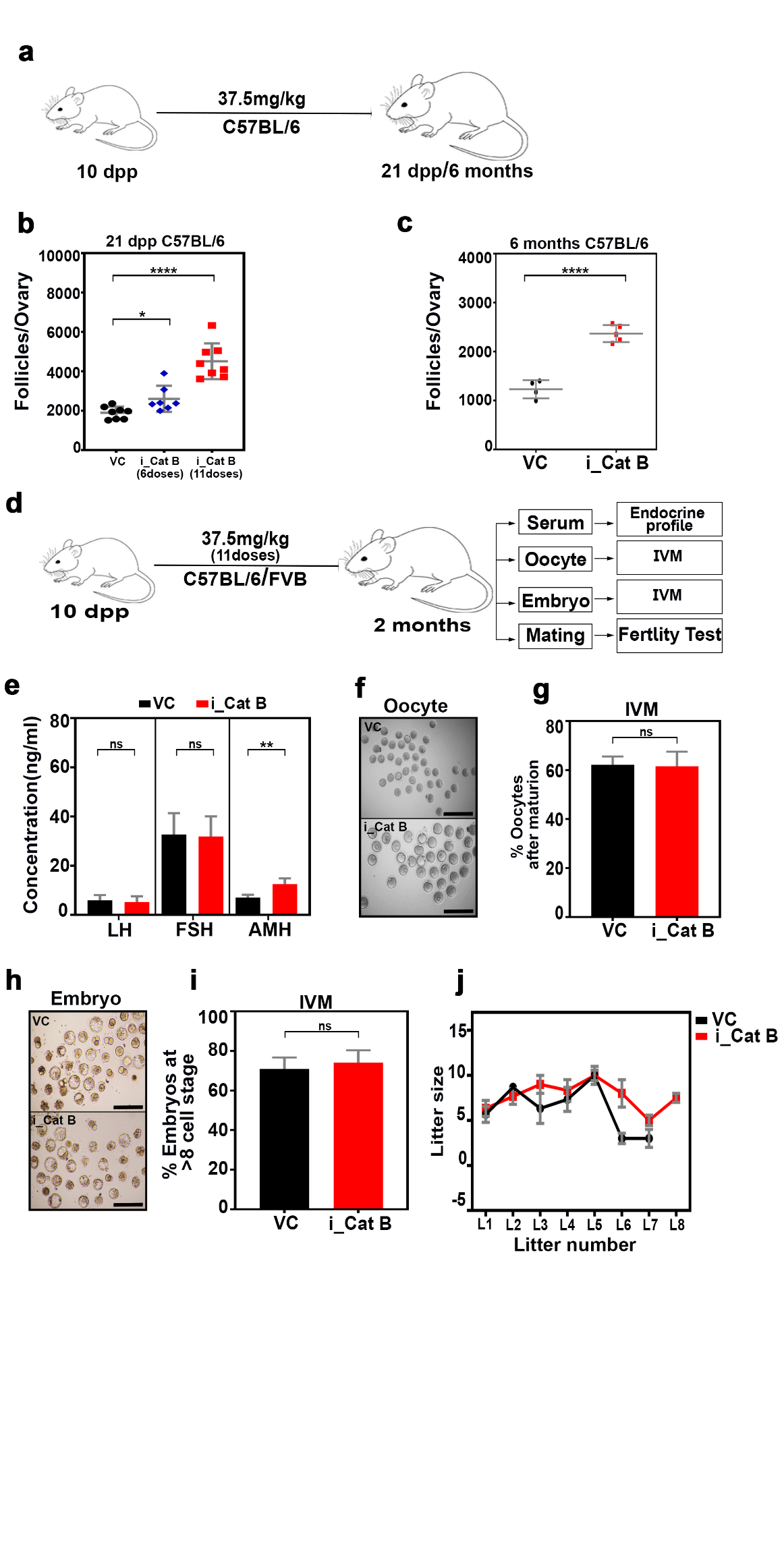


FigS3:


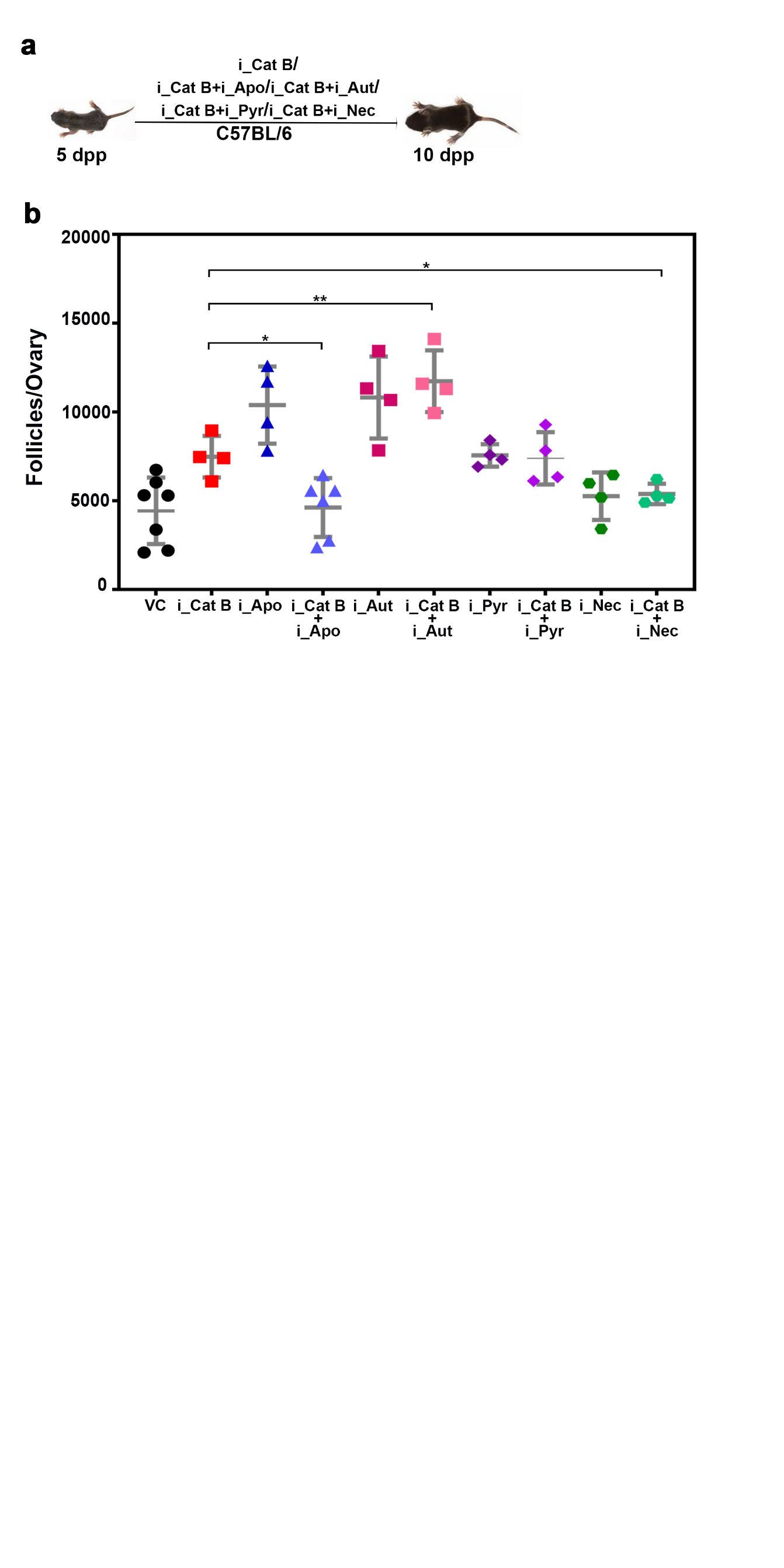


Fig S4:


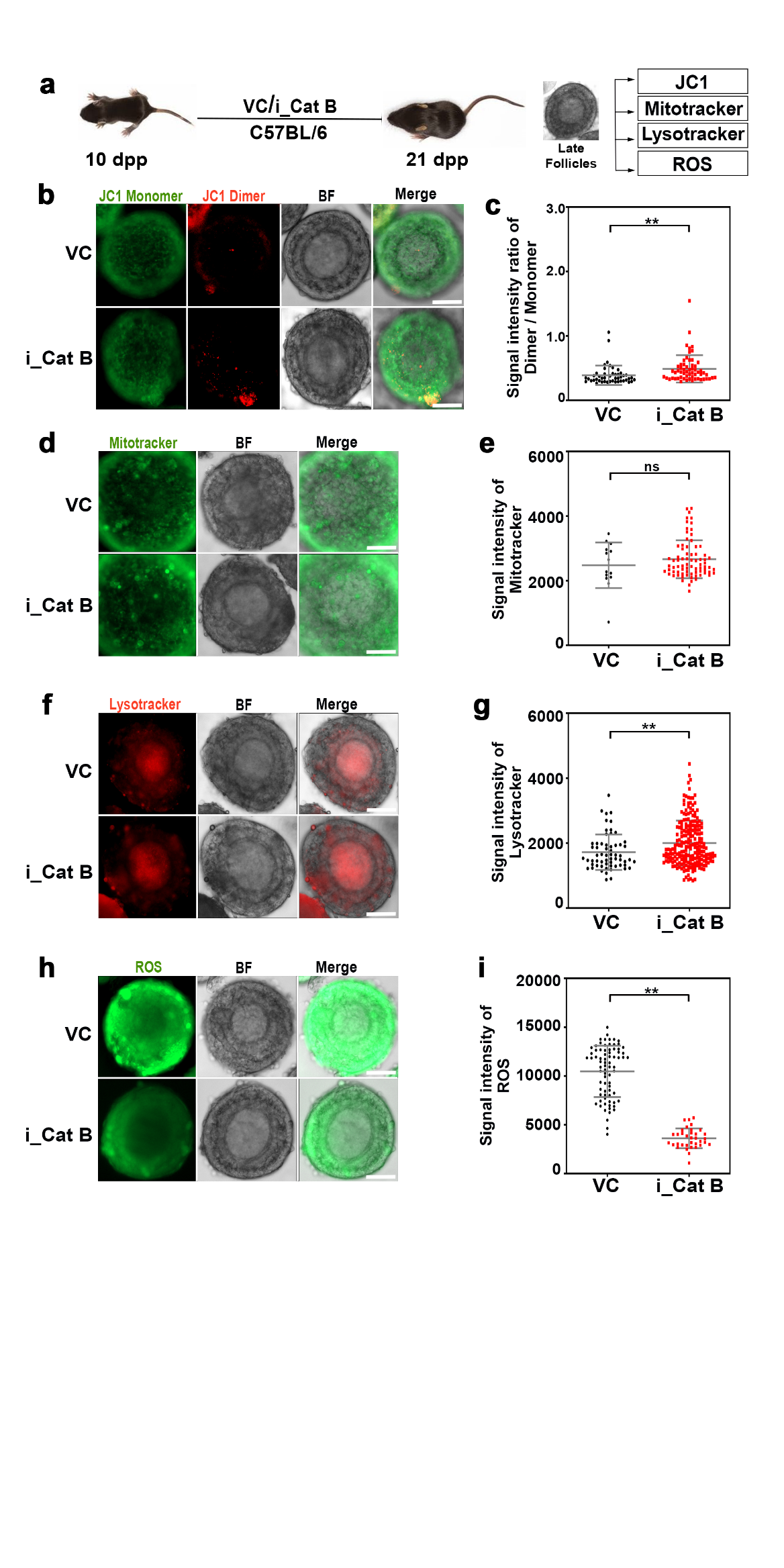


FigS5:


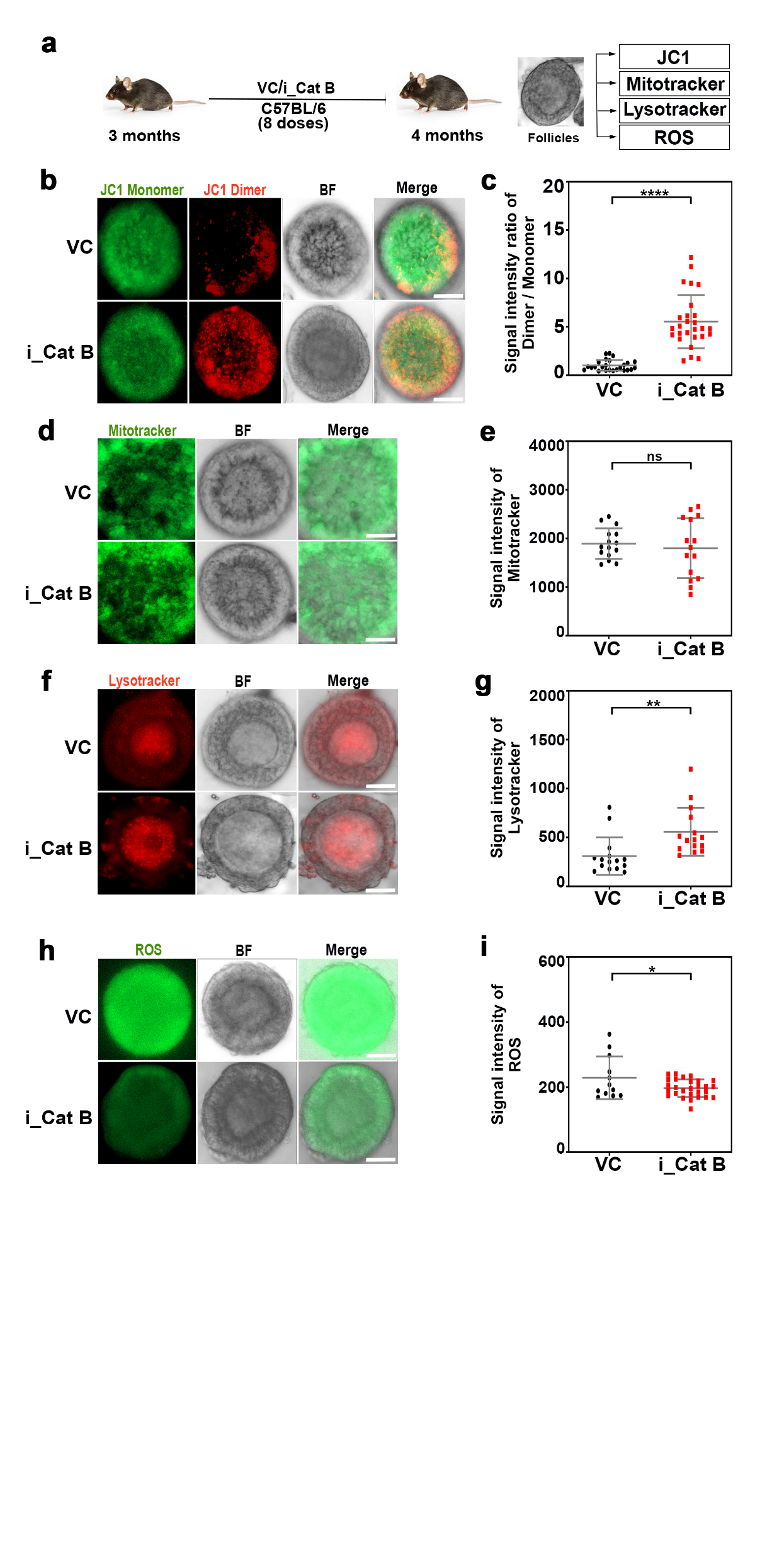


FigS6:


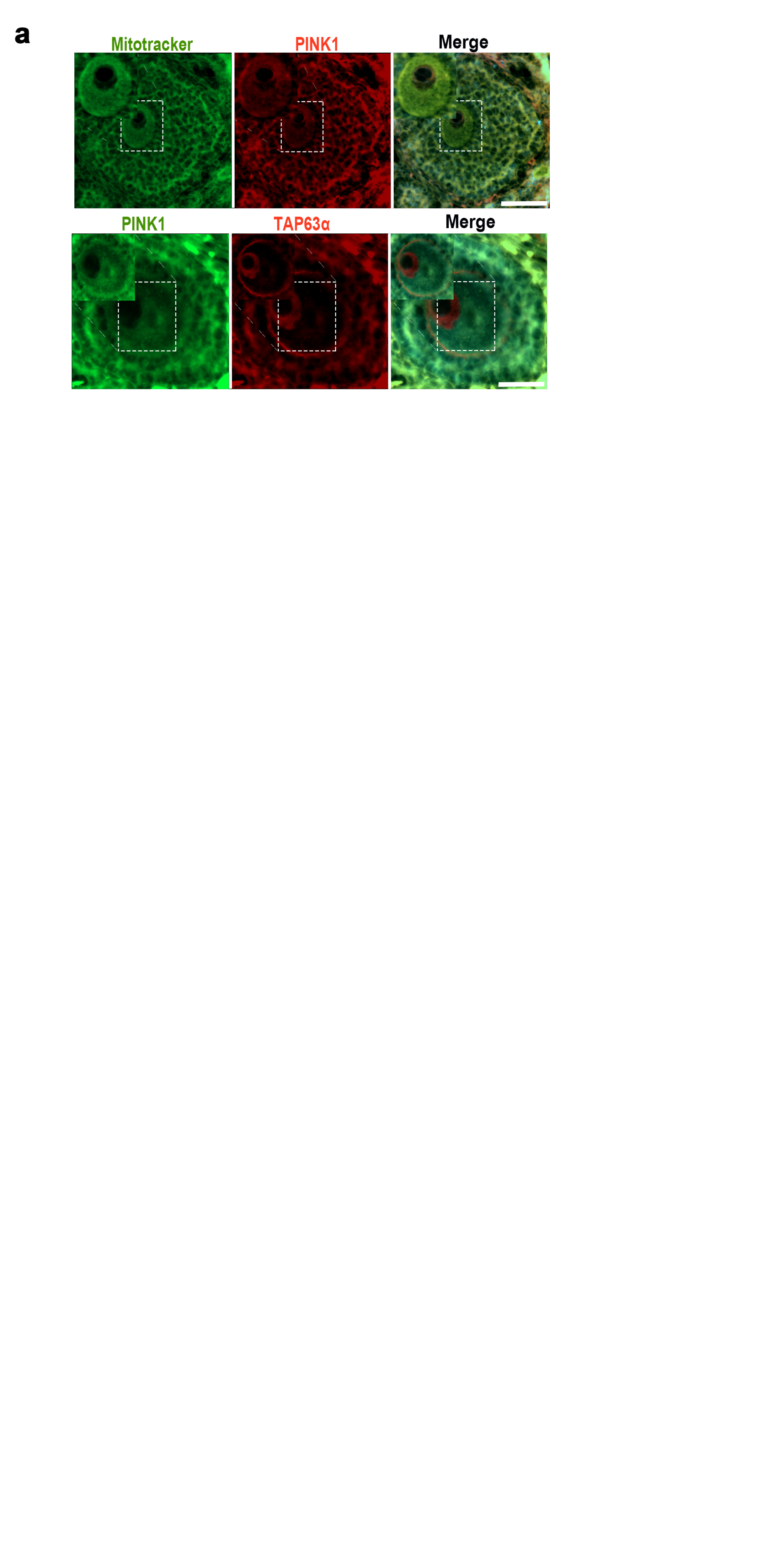


FigS7:


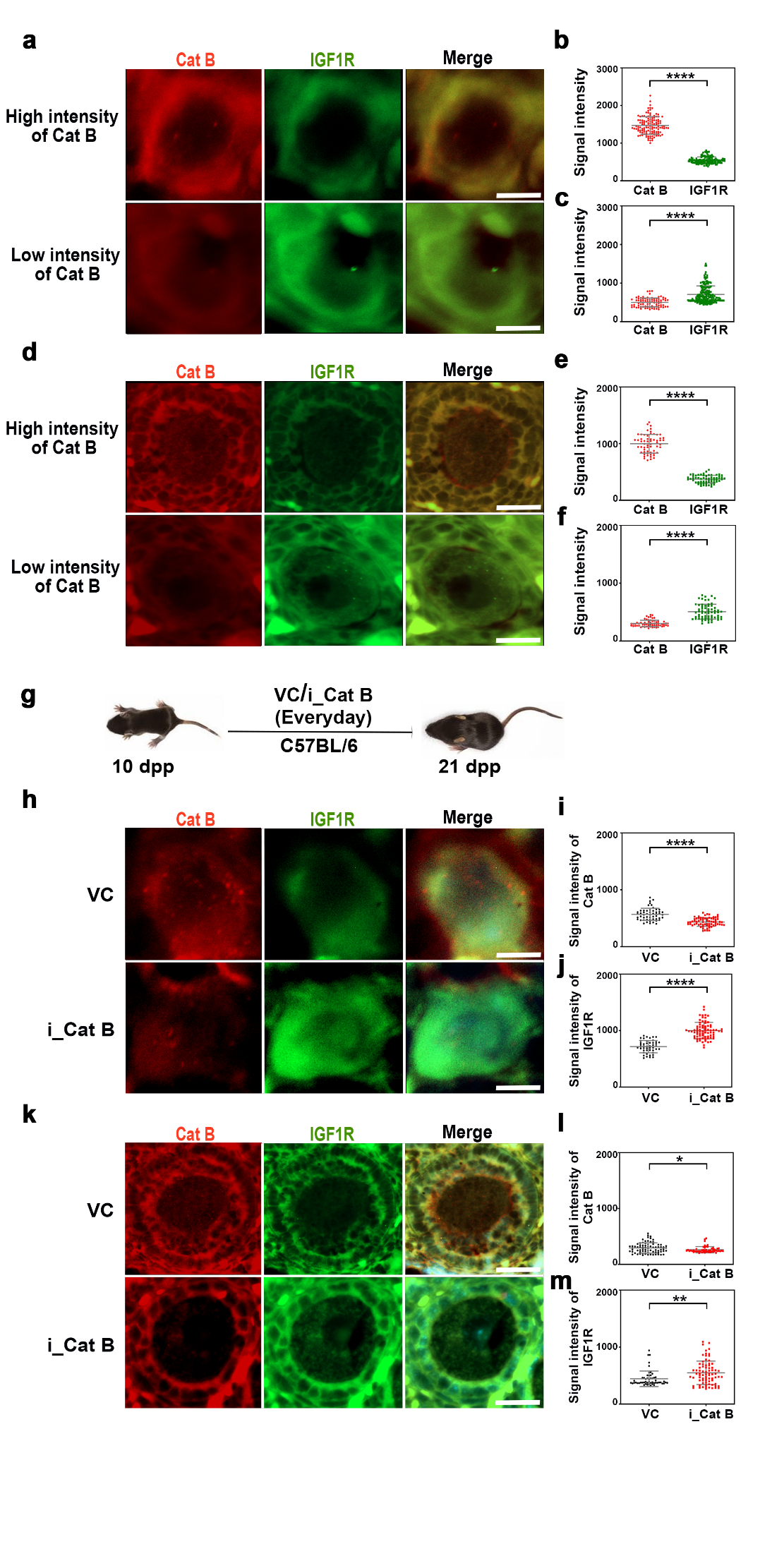


FigS8:


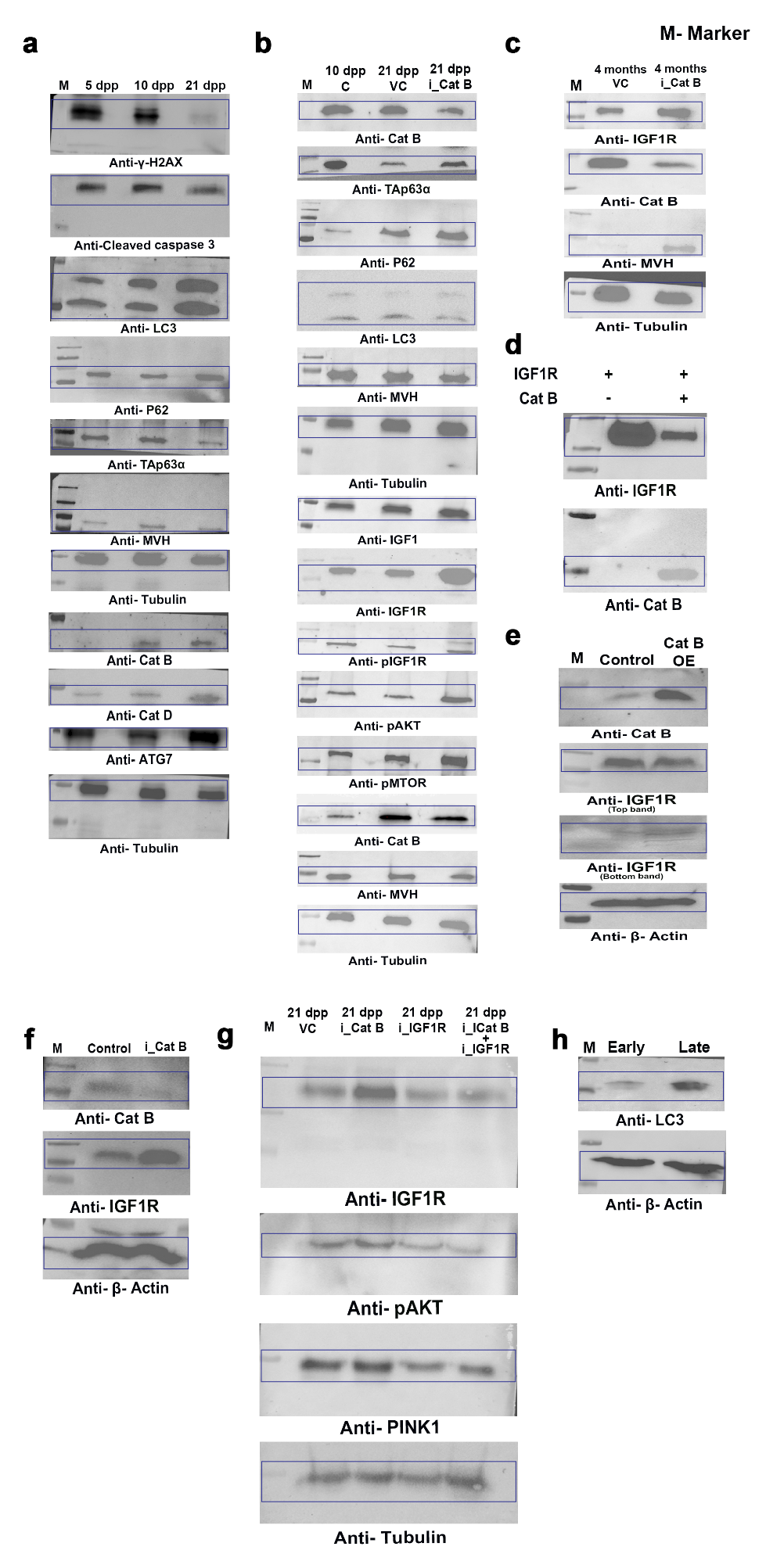

Supplement: Supplementary file 1 — Figure S1. (a–c) Quantification of primordial, primary, and secondary follicles at 10 dpp from VC, i_Apo, i_Aut, i_Pyr, and i_Nec ‐treated mice ovaries. VC, vehicle control; i_Apo, an inhibitor of apoptosis; i_Aut, an inhibitor of autophagy; i_Pyr, an inhibitor of pyroptosis; i_Nec, an inhibitor of necrosis. ****p ≤ 0.0001, ***p ≤ 0.001, **p ≤ 0.004, *p ≤ 0.04, ns ≥ 0.1 unpaired t‐test. Error bars show mean ± SD. Figure S2. (a) Experimental regime for i_Cat B intraperitoneal injection with different doses and in aged mice ovaries (n = 3–5). (b) Follicle counts at 21 dpp with different doses. (c) Follicle counts at 6 months of 11‐dose treatment of VC and i_Cat B mice ovaries, respectively. (d) Experimental regime for i_Cat B intraperitoneal injection followed by endocrine profiling, oocyte, embryo quality assessment, and fertility test experiments. (e) Quantification of LH, FSH, and AMH in the serum from VC‐ and i_Cat B‐treated females. (f) Bright field oocyte maturation images. (g) Quantification of in vitro oocyte maturation from VC and i_Cat B‐treated females. (h) Bright field embryo development images. (i) Quantification of in vitro embryo development from VA and i_Cat B‐treated females. (j) Fertility performance of VC and i_Cat B‐treated females. VC, vehicle control; i_Cat B, an inhibitor of Cathepsin B. ****p ≤ 0.0001, **p ≤ 0.001, *p ≤ 0.02, ns ≥ 0.5 unpaired t‐test. Error bars show mean ± SD. Scale bars are 20 μm. Figure S3. (a) Experimental regime for i_Cat B intraperitoneal injection with different death pathways inhibitors (n = 3–4). (b) Follicle counts at 10 dpp from VC and i_Cat B alone or along with i_Apo‐, i_Aut‐, i_Pyr‐, and i_Nec‐treated females. VC, vehicle control; i_Cat B, an inhibitor of Cathepsin B; i_Apo, an inhibitor of apoptosis; i_Aut, an inhibitor of autophagy; i_Pyr, an inhibitor of pyroptosis; i_Nec, an inhibitor of necrosis. **p ≤ 0.006, *p ≤ 0.01, ns ≥ 0.5 unpaired t‐test. Error bars show mean ± SD. Nonsignificant p values are not repre [file ACEL-24-e70066-s001.docx]
